# The Gambling Behaviour and Attitudes to Sports Betting of Sports Fans

**DOI:** 10.1007/s10899-021-10101-7

**Published:** 2022-02-01

**Authors:** Emma Seal, Buly A. Cardak, Matthew Nicholson, Alex Donaldson, Paul O’Halloran, Erica Randle, Kiera Staley

**Affiliations:** 1 Social and Global Studies Centre, RMIT University, 360 Swanston Street, Melbourne, VIC 3000 Australia; 2 School of Business, La Trobe University, Melbourne, VIC Australia; 3 Monash University Malaysia, Kuala Lumpur, Malaysia; 4 Centre for Sport and Social Impact, La Trobe University, Melbourne, VIC Australia

**Keywords:** Sports betting, Fan behaviour, Sports fans, Gambling harm, Normalisation, Gender

## Abstract

Survey responses from a sample of nearly 15,000 Australian sports fans were used to study the determinants of: (i) gambling behaviour, including if a person does gamble and the type of gambling engaged with; (ii) the number of sports and non-sports bets made over a 12-month period; and (iii) attitudes towards betting on sports. The probability of betting on sports decreased with increasing age and was lower for women and people with a university education. This gender difference varied with age, with the greatest difference found among the young. Similar effects were observed for the number of sports bets made, which declined with age. The gender difference in the number of sports bets also varied with age with the greatest difference found among the young arising from the high propensity of young men to bet on sports. Attitudes to sports betting were also analysed, with a key finding that, within friendship circles, the views that sports betting is perceived as harmless, common and very much a part of enjoying sports were stronger among young men. These permissive attitudes were stronger among people who bet on sports and those who bet on sports more frequently. The analysis of sports fans provides insights into the characteristics of the target market most likely to bet on sports, which can be used to inform public health initiatives and harm reduction campaigns.

**Supplementary Information:**

The online version contains supplementary material available at 10.1007/s10899-021-10101-7.

## Introduction

Harm from gambling is a significant global public health issue, with negative impacts on the health and wellbeing of individuals, families and communities (Gainsbury et al., 2014). Researchers have argued the harm to health and wellbeing caused by gambling is equivalent to that associated with major depressive disorders, and substance misuse and dependence (Browne et al., 2016). There is an array of research linking harmful gambling to health and social issues, including an individual’s health and wellbeing (Rockloff et al., 2020; Suomi et al., 2014), impacts on families and relationships (Dowling, 2014), and an association with intimate partner and family violence (Dowling et al., 2019). Generally, harms related to gambling reflect social and health inequalities, with negative effects unequally skewed towards economically and socially disadvantaged groups (Cowlishaw et al., 2016; Raybould et al., 2021; Wardle et al., 2018). Further, Deans et al., (2017a, 2017b) argued that older adults, young men, and children are most vulnerable to harm from gambling.

In this paper, we explore the gambling choices of a diverse group of sports fans from Victoria, Australia, aged 18 and over, based on data from a survey of almost 15,000 members and fans of elite sporting clubs. In doing so, we investigate the relationship between individual demographic characteristics, the gambling behaviour of these sports fans and differences in attitudes to sports betting. Australia is recognised globally as having one of the most accessible and liberalised gambling environments, with policy and regulation, online platforms and the diversification of gambling products all increasing the availability and uptake of different gambling opportunities (Deans et al., 2016a; Hing et al., 2017; Pitt et al., 2017a). However, this trend is reflected elsewhere, with similar issues reported in the United Kingdom (McGee, 2020), Spain (Lopez-Gonzalez, et al., 2020) and Ireland (Fulton, 2017), implying our analysis is of international importance for those seeking to understand gambling choices and attitudes, and mitigate harm through appropriate policies and programs.

Recent evidence from the Household, Income and Labour Dynamics in Australia survey (a nationally representative longitudinal survey) demonstrated that there were 6.8 million regular gamblers in 2015, of whom an estimated 1.1 million were at risk of harm from gambling-related problems (Armstrong & Carroll, 2017). The National Australian Gambling Statistics Report highlighted that total gambling losses rose 5% between 2017 and 2018 to $24.89 billion. These statistics demonstrate gambling is an ongoing and increasing threat to individual and public health. Not only are individuals at risk of harm from gambling, for one person with problematic behaviour, an estimated five to ten people are adversely affected (Productivity Commission, 1999), implying widespread economic and social costs of gambling (Wardle et al., 2018).

### Rise and Normalisation of Sports Betting

This research focuses on sports betting, a rapidly emerging sector of the gambling industry. Its impact on normalising gambling, especially among the young, has been of increasing concern over the last decade in countries like Australia and the United Kingdom (Purves et al., 2020). Sports betting is one of the few forms of gambling that has shown a substantial increase in participation in recent years (Hare, 2015). In Australia, sports betting resulted in the largest year-on-year percentage increase (16.3%) in gambling losses during 2017–2018. The relationship between sports and gambling is increasingly symbiotic, with teams from Australia’s two major professional sports, the Australian Football League (AFL) and National Rugby League (NRL), significantly involved in the ownership and promotion of gambling products and services. Activities include formal sports partnerships, uniform naming rights, stadium signage and the promotion of odds during televised broadcasts. This general trend has been termed the ‘gamblification’ of sports by McGee (2020) and has become ubiquitous across a variety of sports settings, from elite to community level.

As a consequence of the pervasiveness of sports betting, researchers have increasingly sought to identify and describe the ‘normalisation’ effect of sports betting and its acceptance as part of peer-based socialisation and general sports fandom (Bunn et al., 2019; Raymen and Smith, 2017). A growing body of evidence has started to address the factors that lead to sports betting being perceived as an everyday part of sports, fostering its uptake. This is aligned with an increasing focus within broader gambling research on the influence of the environment and social determinants on people’s behaviour, as opposed to concentrating on a problem or pathology within the individual (Johnston and Regan, 2020).

There has been a strong research focus on the rise and prominence of sports betting marketing, quantifying how prevalent gambling promotions are during sports broadcasting (Milner et al., 2013), on social media platforms (Thomas et al., 2018), in live events within stadia (Thomas et al., 2012), and exploring how online platforms have been harnessed by wagering companies to encourage consumption (Deans et al., 2016a). Thomas et al. (2012) highlighted there were very few visible or audible messages to counter overwhelmingly positive messages about sports betting during matches. Their research also addressed how sports betting advertising and associated strategies affect the attitudes of specific community sub-groups, including young people, parents, and young males. Pitt et al. (2016a) found children could recall sports betting brand names, places they had seen betting advertising and associated plot details of advertisements. Deans et al. (2017b) conducted similar work with young men and demonstrated sports betting marketing influences their betting behaviour.

Research has also focused on people’s attitudes to sports betting advertising, to improve understanding of community sentiment. Generally, this has shown that both parents and young people disagree with the increase in sports betting advertising and have concerns about how these messages promote a seemingly natural affinity between gambling and sports (Nyemcsok et al., 2021; Pitt et al., 2016b). However, Pitt et al. (2016b) reported that young people’s discourses about sports increasingly involve discussions about gambling ‘odds’ and that some young people believe that gambling is a usual and valued consumption activity during sports. Alternative evidence suggests young men feel particularly overwhelmed and bombarded by sports betting advertising (Thomas et al., 2012). This is unsurprising because this group is the target market for most Australian wagering operators. Hing et al. (2016) argued that such operators deliberately position sports betting as an activity engaged in by single, professional, upwardly mobile young men.

Other environmental or ‘normalisation’ issues investigated in the research literature include the availability and convenience of sports betting on mobile phone apps or online, with ease of access facilitating gambling (McGee, 2020), the socio-cultural alignment between sports betting and sports (Deans et al., 2017a; Thomas, 2014), and how physical and online environmental factors influence the gambling risk behaviours of young men (Deans et al., 2016b). For example, Deans et al. (2017a) conducted semi-structured interviews with a convenience sample of 50 Australian men, aged 20–37, who were fans of and had bet on either NRL or AFL matches (games). These young male sports bettors reported their betting was normal and socially accepted, especially among sports fans, and ‘gambling-related language had become embedded in peer discussions about sport’ (p. 112). As such, Deans et al. concluded an exaggerated normalisation of wagering might exist in male sports fans’ peer groups. The young men in their study had established rituals (e.g. punters’ clubs) that reinforced their social connection to sports betting, but also enhanced the peer pressure to bet—an outcome that is perhaps inevitable given the role of social interaction in normalising behaviour (Russell et al., 2018).

### Understanding ‘Sports Bettors’

A separate but connected branch of research literature has concentrated on profiling groups most at risk of experiencing harm from sports betting, particularly describing their attitudes and characteristics. As indicated previously, two groups of major concern are men and youth in general (both male and increasingly female). Studies and reviews have consistently found that young adult males are at greater risk of problem gambling (Hing et al., 2015; Williams et al., 2012). Recently, such research has also explored sports betting specifically. For example, Hing et al. (2016) in a quantitative study with a purposive sample of 639 Australian adults, identified key demographic risk factors for problem sports bettors included being male, younger, never married, and living either alone, in a one-parent family with children, or in a group household. Other risk factors included having a higher level of education and working or studying full-time. Numerous, frequent, and larger bets appeared to characterise high-risk sports bettors, as opposed to those deemed at low risk of experiencing harm. This is supported by recent research by Ayandele, Popoola and Obos (2019), who surveyed 749 Nigerian tertiary students aged 16–30 years to explore how socio-demographic factors, peer-based gambling and sports betting knowledge interact to shape young adults’ attitudes to sports betting. They found a favourable attitude towards sports betting was associated with being older and male, having a knowledge of sports betting and was positively related to the betting attitudes and behaviours of friends.

Whilst understanding normalisation factors and processes is important, alongside the attitudes and characteristics of different sub-groups, what is missing from current research is a large-scale examination of the attitudes of *sports fans* specifically, and the key demographic factors that are associated with their sports betting behaviour. This study addresses the gap. To the best of our knowledge, this research, conducted with almost 15,000 unique respondents, is the largest quantitative study operating at the nexus of sports fans and gambling behaviour. Arguably, the issues described above are more impactful in this cohort because they are highly engaged with sports and very exposed to marketing and the gambling economy. It is imperative to understand how such environmental and socio-cultural processes influence sports fans’ betting behaviour and to identify the sub-groups where these behaviours are most apparent.

Using quantitative research with a broad demographic group of sports fans (aged 18 and over), we aimed to compare attitudes between sports bettor, non-sports bettor and non-bettor cohorts, and examine the factors that would make it more likely for a sports fan to be a sports bettor. Broadly, we focused on attitudes to betting in sports, the risks associated with sports betting and perceptions about how much of a social norm this activity is. The research was guided by the following questions:

1. What demographic factors make it more or less likely for a sports fan to bet on sports? How does this correlate with the number of sports bets a person makes?
2. What is the impact of described normalisation processes on the attitudes of sports fans that bet on sports, compared with those that do not bet at all, and those that bet but not on sports?

Understanding the demographic profiles and risk factors for sports bettors and their attitudes is an increasingly important area of research. The results from this research could inform public health interventions and policy to help ensure they appropriately address areas of concern.

## Data

The research questions above are addressed as part of a broader project undertaken in partnership with the Victorian Responsible Gambling Foundation (VRGF). The project involved a survey that was distributed in collaboration with 17 professional sporting clubs from Australian Football, Basketball, Cricket, Soccer, Netball and Rugby Union in the state of Victoria, Australia. The survey was targeted at members, fans, and supporters of these clubs. In each instance, the survey was shared via social media channels (including Facebook and Twitter) and electronic direct marketing using email to each club’s membership base. Depending on the sporting code and relevant season (summer or winter), the survey was shared either between 30th October–3rd December 2020 or 25th February–18th March 2021. Data were collected in the context of the COVID-19 pandemic and research has demonstrated an increase in sports betting and a decrease in other types of gambling (e.g., casino, horse racing, pokies, etc.) during this period (Jenkinson et al., 2020). Whilst this could have impacted the behaviour and attitudes of respondents in this survey, the results still highlight the groups most at risk from engaging in sports betting and their associated attitudes. The survey took on average 15 min to complete and elicited a total of 17,228 responses. However, due to incomplete survey responses, the estimating sample is restricted to at most 14,950 observations in the analysis below. Three components of the survey were used in this study. First was data on gambling behaviour comprising responses about (i) whether an individual gambles or not and whether any gambling is sports betting, non-sports betting or both; and (ii) the number of bets in a given period.

Participants were asked about gambling activity in general first. They were advised: *“Gambling includes activities in venues such as casino table games, pokies, TAB, Keno *etc*., as well as raffles, lotteries and scratchies. It also includes gambling online or *via* apps such as sports betting, race betting and online pokies and casino games, where you bet with money.”*

This was followed with the questions:

1. Thinking of all these types of gambling, in the past 12 months, have you spent any money on these gambling activities?
2. In the past 12 months, how often have you gambled? (with a number of times per week, month or year options available)

Participants were then asked about sports betting activity. They were advised: “*Sports betting refers to legal wagering with bookmakers on approved types of local, national or international sporting activities, (other than horse or greyhound racing both on or off the course) in person, *via* the telephone or *via* an app or online.”*

This was followed with the questions:

1. Thinking of all these types of sports betting, in the past 12 months, have you spent any money on these sports betting activities?
2. In the past 12 months, how often have you taken part in sports betting? (with a number of times per week, month or year options available)

This data are summarised in the first six rows of Table 1 under the sub-headings ‘betting category’ and ‘number of bets’. For clarity, when referring to the specific participant groups involved in the research, we will use the terms sports bettors, non-sports bettors, or non-bettors to avoid referring to gambling and betting interchangeably. Table 1 shows that about 35% of the sample are non-bettors while another 35% are non-sports bettors (i.e. they bet on lotteries, raffles, poker or slot machines and casino gambling). The remaining 30% of the sample are sports bettors divided evenly between those that engage in sports betting only and those that engage in both sports and non-sports betting. The average number of bets in a year for the full sample is 19.6 for non-sports and 14.5 for sports bettors.^1^ This data are also presented by betting category, showing that sports bettors, on average bet many more times per year than non-sports bettors. However, those that bet on both have a much higher betting frequency again, betting nearly twice as often as those who bet only on sports. The much higher standard deviation among sports bettors is also notable, suggesting there is much greater variation in betting frequency among sports bettors than non-sports bettors.

**Table 1:** Descriptive statistics of survey respondents for the full sample and by the different types of bettors. Full sample includes people who responded that they did not gamble at all. Standard deviations provided in parentheses only for variables that are not dichotomous

Variable	Means			
	Full Sample	Non-Sports Bettors	Sports Bettors Only	Sports + Non-Sports Bettors
*Betting category*				
No betting	0.353	–	–	–
Non-sports betting	0.346	1.000	–	–
Sports betting only	0.154	–	1.000	–
Sports and non-sports betting	0.147	–	–	1.000
*Number of bets*				
Non-sports bets	19.637 (58.845)	34.346 (59.103)	0.000	63.221 (121.478)
Sports bets	14.493 (72.877)	0.000	56.058 (95.032)	41.323 (155.810)
Male	0.694	0.637	0.825	0.786
Age	50.664 (14.769)	55.034 (13.090)	43.283 (13.824)	46.482 (13.825)
Regional	0.262	0.284	0.239	0.258
*Marital Status*				
Single	0.171	0.139	0.206	0.188
Defacto (live together)	0.128	0.106	0.189	0.165
Defacto (live apart)	0.033	0.024	0.049	0.045
Married/Civil Union	0.598	0.644	0.519	0.542
Separated/Divorced	0.050	0.061	0.034	0.046
Widowed	0.021	0.026	0.004	0.014
Parent	0.415	0.404	0.450	0.454
*Education*				
Less than high school	0.129	0.152	0.102	0.121
Completed high school	0.153	0.143	0.182	0.191
TAFE or trade certificate	0.220	0.234	0.229	0.246
University	0.498	0.471	0.487	0.443
*Employment*				
Self employed	0.133	0.137	0.133	0.140
Employed (wage/salary)	0.608	0.568	0.730	0.691
Unemployed	0.022	0.018	0.021	0.031
Home duties	0.014	0.018	0.009	0.006
Student	0.024	0.008	0.032	0.023
Retired	0.188	0.243	0.067	0.098
Unable to work	0.010	0.009	0.008	0.010
*Income*				
Less than $10 k	0.011	0.008	0.009	0.007
$10 k–less than $20 k	0.014	0.013	0.008	0.014
$20 k–less than $30 k	0.029	0.029	0.019	0.024
$30 k–less than $40 k	0.032	0.039	0.022	0.023
$40 k–less than $50 k	0.039	0.044	0.031	0.031
$50 k–less than $60 k	0.049	0.052	0.047	0.043
$60 k–less than $80 k	0.095	0.095	0.098	0.105
$80 k–less than $100 k	0.102	0.103	0.105	0.112
$100 k–less than $125 k	0.101	0.095	0.115	0.109
$125 k–less than $150 k	0.087	0.082	0.098	0.098
$150 k–less than $200 k	0.117	0.107	0.144	0.141
$200 k or more	0.121	0.107	0.152	0.148
Prefer not to say	0.203	0.225	0.154	0.144
*Country of birth*				
Australia	0.934	0.936	0.940	0.946
UK or New Zealand	0.058	0.058	0.054	0.049
Other	0.008	0.006	0.006	0.005
*Parents’ country of birth*				
Both in Australia	0.677	0.689	0.670	0.682
One in Australia	0.143	0.129	0.157	0.165
Both overseas	0.180	0.181	0.173	0.153
Aboriginal Torres Strait Islander	0.008	0.007	0.010	0.010
Health condition or disability	0.108	0.119	0.079	0.095
Observations	14,813	5120	2285	2178

Observations for Non-Sports Bets and Sports Bets variable are 14,293 and 14,686 respectively for the full sample. For Non-Sports bettors only, observations for Non-Sports Bets are 5102. For Sports bettors only, observations for Sports Bets are 2285. For Sports + Non-Sports bettors, observations for Non-Sports Bets and Sports Bets are 1658 and 2051 respectively

The second component of the data used in this study comprises demographic characteristics. This includes gender, age, location (metropolitan or regional), marital status, education, employment status, income categories, country of birth, parents’ country of birth, Aboriginal Torres Strait islander origin and health status. The data are summarised for the full sample and the different betting categories in Table 1. Some key features of the data are that about 70% of the sample is male but nearly 80% of sports bettors are male and the average age of the sample is 50 years, but sports bettors have an average age of 43 years for sports bettors and 46 years for those who both sports and non-sports bet. Two other notable features are the much higher proportion of non-sports bettors who are retirees (0.24) compared to the proportion of sports bettors (0.07) and the high proportion of responders who did not report their income (0.20).

The third component of the data used here comprises responses to questions about attitudes to gambling. The questions are grouped into two categories including (i) general attitudes to gambling and sports betting; and (ii) perceptions of the attitudes and behaviours of others. Responses were elicited on a scale from 0 (totally disagree) to 10 (totally agree). The questions are presented in Table 2, where sample means and standard deviations are presented for the full sample and the different betting categories.

**Table 2:** Summary statistics of responses to questions about gambling for the full sample and by the different types of bettors. Standard deviations in parentheses

Question	Mean and Standard Deviation				
	Full Sample	Non-Bettors	Non-Sports Bettors	Sports Bettors Only	Sports + Non-Sports Bettors
*General attitudes to sports betting*					
Sports betting should not be part of experiencing sport	6.845 (3.238) [0.000]	7.813 (2.970)	7.221*** (3.080)	5.267*** (3.160)	5.292*** (3.124)
People who bet regularly on sport are at risk of harm from gambling	7.221 (2.519) [0.000]	7.795 (2.375)	7.296*** (2.469)	6.497*** (2.571)	6.426*** (2.525)
Sports betting can place people at higher risk of relationship problems, mental health and wellbeing issues and money worries	8.028 (2.320) [0.000]	8.511 (2.173)	8.087*** (2.275)	7.486*** (2.333)	7.298*** (2.446)
Regular discussion of the ‘odds’ when talking about sport can lead to gambling problems in individuals	6.317 (2.717) [0.000]	6.707 (2.734)	6.386*** (2.659)	5.870*** (2.655)	5.688*** (2.695)
It’s easy for people with sports betting issues to stop gambling	1.973 (2.456) [0.000]	1.677 (2.421)	1.873*** (2.427)	2.396*** (2.433)	2.477*** (2.498)
*Perceptions of other’s attitudes and behaviours*					
Most people in society think betting on sport is harmless	4.489 (2.600) [0.000]	4.513 (2.774)	4.337*** (2.602)	4.642 (2.354)	4.627 (2.383)
Most people in society bet on sport	3.831 (2.423) [0.000]	3.766 (2.511)	3.513*** (2.388)	4.415*** (2.270)	4.124*** (2.305)
Most people in my family think betting on sport is harmless	3.586 (2.862) [0.000]	2.875 (2.854)	3.374*** (2.808)	4.584*** (2.628)	4.744*** (2.581)
Most people in my family bet on sport	1.707 (2.427) [0.000]	1.160 (2.101)	1.331*** (2.134)	2.771*** (2.722)	2.784*** (2.754)
Most people in my friendship group think betting on sport is harmless	4.483 (2.827) [0.000]	3.814 (2.880)	4.128*** (2.750)	5.732*** (2.463)	5.616*** (2.446)
Most people in my friendship group bet on sport	3.456 (3.030) [0.000]	2.562 (2.742)	2.735*** (2.700)	5.487*** (2.850)	5.166*** (2.886)
Odds talk is common in discussions about sport with my friends and peers	3.299 (3.100) [0.000]	2.687 (2.989)	2.633 (2.861)	4.911*** (3.006)	4.641*** (2.951)
Observations	14,813	5,230	5,120	2,285	2,178

Means of each gambling group are compared to non-gamblers using a *t*-test with a Sidak correction to account for repeated testing, with the level of statistical significance denoted by * (10%), ** (5%) and *** (1%). *P*-values of an *F*-test of whether mean responses of each group are statistically significantly different from each other presented in Full Sample column in braces

An important research question here is whether people in different betting categories respond to each question differently. We conducted a one-way analysis of variance (ANOVA) for each question to test whether the mean responses of each group are statistically significantly different. The *p*-value of this joint *F*-test for each question is presented in the ‘Full Sample’ column in braces; all tests have *p*-value of [0.000] implying we reject the null hypothesis of equality between the mean response of each group to each question. We also test the differences between means for each group using *t*-tests with a Sidak correction to account for the possibility of a false positive finding given the large number of *t-*tests. The table reports results of tests of differences between the mean response of non-bettors and each type of betting group with statistically significant differences at the 10%, 5% and 1% levels denoted by *, ** and *** respectively. The results show average responses of almost all betting groups differ from those of non-betters at the 1% level of significance. Differences are most stark in relation to the notion that ‘sports betting should not be part of experiencing sport’ and items related to the social aspects of sports betting, particularly the place of sports betting in the person’s family and friendship groups. However, responses to (i) ‘most people in society think betting on sport is harmless’ were not statistically different between sports bettors and non-bettors; and (ii) ‘odds talk is common in discussions about sport with my friends and peers’ were not statistically different between non-bettors and non-sports bettors.

An important feature of the survey is that it reached beyond people who identify as gamblers, providing insights into a more diverse sample than many previous studies. However, a limitation is that the design is focused on members, fans or supporters of a group of elite clubs or teams, implying to some degree that respondents are likely to be more engaged with sport than the average member of the Victorian population. Therefore, the differences between bettors and non-bettors identified here are potentially lower bounds and analysis of a more representative sample might uncover even greater differences. Another important benefit of the sampling frame is that it is likely the target audience for sports betting advertisers. Therefore, the analysis offers insights into a group that is likely most targeted and affected by sports betting advertising and understanding this group provides valuable insights to harm minimization policies with respect to sports betting.

## Empirical Methods

The empirical analysis can be divided into two broad approaches. The first is to analyse the determinants of the betting choices of survey respondents. The second is to analyse the responses to gambling attitude questions. The approaches to these analyses are described in turn below.

### Who Bets and How Often?

Each survey respondent is assumed to choose between four types of betting activity: no betting, non-sports betting, sports betting or both sports and non-sports betting. We define the gambling choice of each survey respondent $i$ as ${G}_{i}=j\in \left\{1, 2, 3, 4\right\}$. We want to understand the relationships between different demographic characteristics’ and gambling choices. Given these four possible gambling choices or outcomes are unordered, we used a multinomial Probit specification to estimate the relationships. The probability that individual $i$ makes gambling choice ${G}_{i}=j$ is given by

1 $$p_{ij} = P\left[ {G_{i} = j\left| { X} \right._{i} } \right] = {\Phi }\left( {X_{i}^{{\prime}} \beta_{j} } \right) j = 1, \ldots , 4,$$

where ${X}_{i}$ is a vector of personal characteristics (including gender, age, location, marital status, education, employment status, income categories, country of birth, parents’ country of birth, Aboriginal Torres Strait islander origin and health status, as listed in Table 1), $\Phi \left(.\right)$ is the cumulative density function of the Standard Normal distribution and ${\beta }_{j}$ provides the parameter estimates on ${X}_{i}$ for gambling choice $j$; see Greene (2018) Chapter 18 for more details. This model allows us to understand the influence of each characteristic on the four possible gambling choices from a model that jointly estimates the probabilities of each alternative gambling choice.

As parameter estimates do not have a clear intuitive interpretation in such models, we compute marginal effects for each ${X}_{i}$ which are given by

2 $$\frac{{\partial p_{ij} }}{{\partial X_{i} }} = \delta_{ij} = p_{ij} \left( {\beta_{j} - \overline{\beta }} \right),$$

where $\overline{\beta }$ is the probability weighted average of the parameter estimate across the four different possible gambling choices. The multinomial Probit results reported in Table 3 below are average marginal effects which are computed as the average of for ${\delta }_{ij}$ across all $i$ individuals. The interpretation of these marginal effects is that they tell us the impact of a unit increase in ${X}_{i}$ (for example female versus male or a one-year increase in age) on the probability of making gambling choice $j$; that is, four marginal effects will be reported for each variable, one for each of no betting, non-sports betting, sports betting or both sports and non-sports betting.

**Table 3:** Results of estimation of multinomial Probit model of betting behaviour. Average marginal effects on each betting behaviour are presented

Variables	Possible Outcomes (Betting Behaviour)			
	Non-Bettor	Non-Sports Bettors	Sports Bettors	Sports and Non-Sports Bettors
Female	0.053***	0.105***	− 0.097***	− 0.061***
	(0.009)	(0.009)	(0.006)	(0.006)
Age	0.000	0.007***	− 0.005***	− 0.002***
	(0.000)	(0.000)	(0.000)	(0.000)
Regional	− 0.014	0.022**	− 0.007	− 0.001
	(0.009)	(0.009)	(0.007)	(0.007)
*Marital Status*				
Defacto (live together)	− 0.027*	0.013	0.009	0.005
	(0.015)	(0.015)	(0.011)	(0.011)
Defacto (live apart)	− 0.042*	0.022	− 0.003	0.023
	(0.023)	(0.025)	(0.017)	(0.018)
Married/Civil Union	0.028**	0.009	− 0.018*	− 0.019*
	(0.013)	(0.013)	(0.010)	(0.010)
Separated/Divorced	− 0.015	0.019	− 0.013	0.009
	(0.021)	(0.020)	(0.017)	(0.017)
Widowed	0.062*	− 0.007	− 0.082***	0.027
	(0.032)	(0.029)	(0.023)	(0.028)
Parent	− 0.003	0.004	− 0.000	− 0.002
	(0.010)	(0.010)	(0.007)	(0.007)
*Highest Education*				
Completed High School	− 0.012	− 0.007	0.001	0.018
	(0.014)	(0.015)	(0.012)	(0.012)
TAFE or Trade Certificate	0.001	0.018	− 0.013	− 0.006
	(0.013)	(0.013)	(0.011)	(0.011)
University	0.087***	− 0.022*	− 0.026**	− 0.040***
	(0.012)	(0.012)	(0.010)	(0.010)
*Employment (base – Self Employed)*				
Employed (wage/salary)	− 0.006	0.020*	− 0.008	− 0.007
	(0.012)	(0.012)	(0.009)	(0.009)
Unemployed	0.030	− 0.010	− 0.046**	0.025
	(0.029)	(0.028)	(0.019)	(0.023)
Home Duties	0.051	0.057*	− 0.035	− 0.073***
	(0.036)	(0.034)	(0.026)	(0.022)
Student	0.136***	− 0.048	− 0.056***	− 0.032
	(0.035)	(0.035)	(0.019)	(0.022)
Retired	0.054***	0.004	− 0.036***	− 0.021
	(0.017)	(0.016)	(0.013)	(0.013)
Unable to Work	0.118***	− 0.082**	− 0.014	− 0.022
	(0.044)	(0.037)	(0.033)	(0.030)
*Income (base—less than $10,000)*				
$10 k–less than $20 k	− 0.072	0.025	− 0.013	0.059*
	(0.052)	(0.048)	(0.033)	(0.034)
$20 k–less than $30 k	− 0.088*	0.021	0.021	0.046
	(0.046)	(0.043)	(0.030)	(0.029)
$30 k–less than $40 k	− 0.153***	0.081*	0.036	0.035
	(0.045)	(0.043)	(0.030)	(0.028)
$40 k–less than $50 k	− 0.131***	0.073*	0.026	0.032
	(0.044)	(0.042)	(0.029)	(0.027)
$50 k–less than $60 k	− 0.122***	0.055	0.036	0.032
	(0.043)	(0.041)	(0.028)	(0.026)
$60 k–less than $80 k	− 0.148***	0.054	0.034	0.061**
	(0.042)	(0.039)	(0.026)	(0.025)
$80 k–less than $100 k	− 0.150***	0.060	0.033	0.058**
	(0.042)	(0.039)	(0.026)	(0.025)
$100 k–less than $125 k	− 0.146***	0.049	0.045*	0.052**
	(0.042)	(0.039)	(0.026)	(0.025)
$125 k–less than $150 k	− 0.155***	0.066*	0.034	0.055**
	(0.042)	(0.040)	(0.027)	(0.025)
$150 k–less than $200 k	− 0.173***	0.063	0.044*	0.066***
	(0.042)	(0.039)	(0.026)	(0.025)
$200 k or more	− 0.184***	0.051	0.057**	0.076***
	(0.042)	(0.039)	(0.027)	(0.025)
Prefer not to say	− 0.119***	0.065*	0.029	0.025
	(0.041)	(0.038)	(0.025)	(0.024)
*Country of birth (base—Australia)*				
UK or New Zealand	0.010	− 0.040**	0.019	0.011
	(0.019)	(0.018)	(0.015)	(0.015)
Other	0.133***	− 0.116***	− 0.003	− 0.014
	(0.047)	(0.039)	(0.035)	(0.035)
*Parents’ Country of birth (base—both Australia)*				
One in Australia	0.007	0.003	− 0.013	0.003
	(0.011)	(0.011)	(0.008)	(0.008)
Both Overseas	0.027**	0.006	− 0.006	− 0.027***
	(0.012)	(0.012)	(0.009)	(0.008)
ATSI	0.021	− 0.009	− 0.003	− 0.008
	(0.044)	(0.043)	(0.030)	(0.030)
Disability or Health Issue	− 0.005	0.008	− 0.004	0.002
(> 6 months)	(0.013)	(0.013)	(0.010)	(0.010)

Model includes linear, second and third order age terms. All age terms interacted with gender indicator. Standard errors in parentheses. Level of statistical significance denoted by * (10%), ** (5%) and *** (1%). Sample size *N* = 14,813

Along with the choice of gambling type, individuals choose how many bets to place, and the factors that influence this number of bets are also of interest. Since 35% of survey respondents are non-bettors, the data on the number of bets comprises a large number of zeros. To accommodate this feature of the data, a Cragg hurdle model is adopted (Cragg, 1971). This model involves two parts: the first is a model of the decision to gamble (selection model), while the second is a model of the number of bets. As we have data on the number of sports bets and non-sports bets, we estimate this two-part model separately for each of these choices. The first part of the model is the selection decision, given by

3 $$C_{i} = \left\{ {\begin{array}{*{20}l} 1 & {{\text{if }}X_{i}^{'} \alpha + \varepsilon _{i} > 0} \\ 0 & {{\text{otherwise}}} \\ \end{array} } \right. , $$

where ${C}_{i}$ is individual $i$’s choice of whether to bet on sports (1) or not (0) or alternatively to make non-sports bets (1) or not (0). The control variables ${X}_{i}$ are as defined above for the multinomial Probit model in Eq. (1), while $\alpha$ is a vector of parameters capturing the influence of each control variable on the decision to place bets or not and ${\epsilon }_{i}$ is a mean zero, constant variance normally distributed disturbance term. The second part of the model estimates the number of observed bets. This continuous variable is given by

4 $$B_{i} = C_{i} \times \exp \left( {X_{i}^{^{\prime}} \theta + u_{i} } \right),$$

an exponential specification of the Cragg hurdle model where ${B}_{i}$ is the number of bets made per year by individual $i$, $\theta$ is the set of parameters reflecting the impact of variables ${X}_{i}$, again as defined above and ${u}_{i}$ is a mean zero, constant variance disturbance term.

The key idea of this model is that the decision to gamble is modelled separately from the decision of how many bets to place. Estimates of $\alpha$ are important determinants of the decision not to gamble and therefore choose zero bets, whereas $\alpha$ and $\theta$ together determine the number of bets if a person chooses to gamble. The overall marginal effect of control variables ${X}_{i}$, which in our specification are common to the selection and number of bets equations, are computed using the *margins* command in STATA and presented in Table 4 below. The detailed expressions for these marginal effects can be found in Burke (2009). The interpretation of these marginal effects is that they reflect the impact of a unit change in the value of a control variable ${X}_{i}$ (i.e. gender or age) on the average number of bets in a given period of time (a year in this instance).

**Table 4:** Models of number of bets (intensity of gambling). Estimates are based on Cragg Hurdle regression. Average marginal effects are presented

Variables	Number of Non-Sports Bets	Number of Sports Bets
Female	− 9.155***	− 18.363***
	(0.944)	(0.885)
Age	0.517***	− 0.441***
	(0.061)	(0.057)
Regional	2.540**	− 1.679*
	(1.091)	(0.973)
*Marital Status*		
Defacto (live together)	− 0.912	1.242
	(2.040)	(1.739)
Defacto (live apart)	1.004	2.424
	(3.437)	(2.804)
Married/Civil Union	− 4.714***	− 4.115***
	(1.718)	(1.544)
Separated/Divorced	0.277	− 1.212
	(2.684)	(2.653)
Widowed	− 2.361	− 4.260
	(3.629)	(4.253)
Parent	− 4.162***	− 0.759
	(1.105)	(1.079)
*Highest Education*		
Completed High School	− 3.421	− 1.187
	(2.296)	(2.294)
TAFE or Trade Certificate	− 6.660***	− 6.765***
	(2.047)	(2.067)
University	− 15.648***	− 9.827***
	(1.863)	(1.974)
*Employment (base—Self Employed)*		
Employed (wage/salary)	2.708**	− 1.891
	(1.337)	(1.456)
Unemployed	4.074	− 0.368
	(3.746)	(3.501)
Home Duties	− 2.920	− 5.631
	(3.262)	(4.111)
Student	− 6.251*	− 8.647***
	(3.313)	(2.391)
Retired	1.131	− 4.668**
	(1.784)	(2.150)
Unable to Work	2.293	− 5.594
	(5.354)	(4.007)
*Income (base—less than $10,000)*		
$10 k–less than $20 k	5.003	2.290
	(5.623)	(4.238)
$20 k–less than $30 k	2.489	6.465
	(4.730)	(4.258)
$30 k–less than $40 k	1.013	1.652
	(4.518)	(3.650)
$40 k–less than $50 k	6.935	9.005**
	(4.721)	(4.173)
$50 k–less than $60 k	4.656	10.202***
	(4.509)	(3.947)
$60 k–less than $80 k	7.698*	7.985**
	(4.379)	(3.394)
$80 k–less than $100 k	7.999*	4.274
	(4.379)	(3.256)
$100 k–less than $125 k	7.566*	8.008**
	(4.398)	(3.385)
$125 k – less than $150 k	4.729	4.461
	(4.376)	(3.318)
$150 k–less than $200 k	6.564	8.874***
	(4.371)	(3.398)
$200 k or more	9.412**	7.710**
	(4.451)	(3.360)
Prefer not to say	3.942	3.103
	(4.194)	(3.170)
*Country of Birth (base—Australia)*		
UK or New Zealand	0.446	2.330
	(2.328)	(2.456)
Other	− 4.848	− 5.933
	(4.937)	(3.699)
*Parents’ Country of Birth (base—both Australia)*		
One in Australia	1.082	− 0.863
	(1.430)	(1.222)
Both Overseas	− 4.281***	− 4.092***
	(1.275)	(1.194)
ATSI	4.063	10.913
	(6.455)	(7.195)
Disability or Health Issue	1.326	1.795
(> 6 months)	(1.591)	(1.768)
Observations	14,297	14,950

Standard errors in parentheses. Level of statistical significance denoted by * (10%), ** (5%) and *** (1%)

### Factors Affecting Attitudes Towards Sport Betting

Our analysis of responses to the 12 different questions about attitudes to sports betting listed in Table 2 comprises two key objectives. First, we are interested in the relationship between individual gambling choices and attitudes to sports betting—this involves the type of betting and the number of bets. It is anticipated that sports bettors will hold more positive attitudes towards sports betting than non-bettors or non-sports bettors. Second, we are also interested in the relationship between demographic characteristics and attitudes to sports betting. Responses to each question range on a scale from 0 to 10 but are standardized to have mean zero and standard deviation of one, to enable comparisons of effects of different variables across survey questions. These standardized responses are modelled using OLS. The model estimated is given by

5 $$A_{i} = Z_{i}^{^{\prime}} \gamma + \varepsilon_{i} ,$$

where ${A}_{i}$ is the response of individual $i$ to one of the 12 questions on their attitude to gambling listed in Table 2. For each question, two versions of the model are estimated where ${Z}_{i}$ is a vector of control variables, including all variables in ${X}_{i}$ which is as defined above, along with either (i) gambling choice, ${G}_{i}$, (no betting, non-sports betting, sports betting or both sports and non-sports betting); or (ii) the number of sports and non-sports bets, ${B}_{i}$, included. Model parameters are given by $\gamma$ while ${\varepsilon }_{i}$ is a disturbance term with zero mean and constant variance. As the dependant variable, ${A}_{i}$, is a standardized measure of responses to attitude questions, $\gamma$ should be interpreted as the average number of standard deviations of change in ${A}_{i}$ per unit change in ${Z}_{i}$.

In all models, we allow for non-linear age effects by including quadratic and higher order age terms, along with a linear age term, testing their significance using a likelihood ratio (LR) test. We also allow for gender effects to vary with age by including age and gender interactions and testing for their significance, again using a LR test.

## Results

### Factors Affecting Gambling Behaviour

**Fig. 1:**
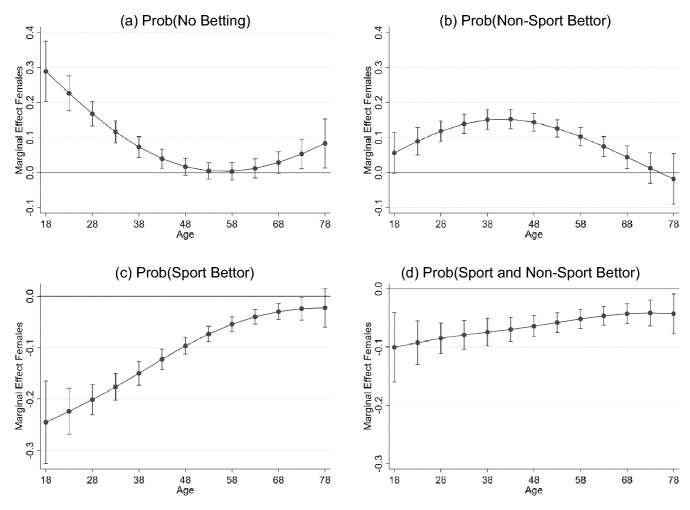
Marginal effect of gender on the probability of each betting category plotted over age. Panels (**a**)–(**d**) are based on estimates presented in columns (1)–(4) of Table 3 respectively. Point estimates are denoted by dots with 95% confidence intervals for each point estimate included

The results of estimation of Eq. (1) are presented in Table 3. The average marginal effects of the listed control variables on different gambling choices of no betting, non-sports betting, sports betting and both sports and non-sports betting are presented in first, second, third and fourth columns respectively. The model includes linear, quadratic and third order age terms.^2^ Focusing on the cases of sports betting only (third column) and both types of betting (fourth column) and on results that are significant at the 1% level (denoted by ***), we found that relative to males, females are 9.6% less likely to bet on sport and 6.2% less likely to bet on both. To test the hypothesis that gender effects vary with age, all the included age terms are interacted with the gender indictor, with these interactions supported by a LR test = 62.63 (*p* value = 0.00). The marginal effect for females relative to males is plotted for each betting category in panels (a)–(d) of Fig. 1. The difference between men and women sports betting is greatest among the youngest in the sample and the difference decreases with age. The result suggests that young men are up to 25% more likely than women of the same age to bet on sports; the difference is less than 10% for people over 50 years. In addition, the average marginal effect of a 1-year increase in age is reported in Table 3. Comparing otherwise identical individuals with a 10-year age difference, the older person is 5.0% less likely to sports bet only and 2.0% less likely to make both sports and non-sports bets relative to the younger person.

Relative to self-employed, which is the base category, students are 6.7% less likely to bet on sports, while those on home duties are 7.0% less likely to gamble on both sports and non-sports. Income results are all relative to the base range of “less than $10,000”, with little difference between different income ranges in the probability of sports betting only. However, respondents above $60,000 are between 5.6 and 8.0% more likely to bet on both sports and non-sports than those in the base income range. This suggests there is little effect on gambling probability of additional income as the marginal effects are similar for each category above $60,000. Finally, respondents whose parents were both born overseas were 2.7% less likely than the base category (both parents born in Australia) to bet on both sports and non-sports.

In Table 4, we present estimates of the model of the number of bets specified in Eqs. (3) and (4). The marginal effects of the full set of control variables on the number of non-sports bets and sports bets are presented in first and second columns of Table 4 respectively. Age is included in these models through linear, quadratic and cubic terms.^3^ As the number of bets is a continuous variable, the marginal effects are interpreted as the impact of a unit change in the control variable on the number of bets per year.

**Fig. 2:**
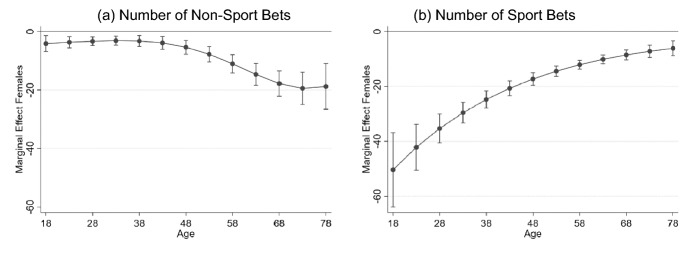
Marginal effect of gender on the (**a**) number of non-sports bets and (**b**) number of sports bets, plotted over age. Panels (**a**) and (**b**) are based on estimates presented in columns (1) and (2) of Table 4 respectively. Point estimates are denoted by dots with 95% confidence intervals for each point estimate included

Focusing first on results significant at the 1% level (denoted by ***) for the number of non-sports bets, females make on average 9 fewer bets per year than males. Betting increases with age, with a 10-year older person making on average 5.29 more bets. The model also includes an interaction between all age terms and gender to test the hypothesis that gender effects vary with age — this interaction is supported relative to the model without the interactions, LR test = 28.41 (*p* value = 0.00). The marginal effect of gender on the number of non-sports bets increases with age with women over 58 placing around 20 fewer bets than men of the same age: panel (a), Fig. 2. Further results include that someone who is married makes 4.5 fewer bets than a single person and parents place 5.0 fewer bets than people with no children. Education reduces betting with those holding trade qualifications betting 6.3 times less than someone who did not complete high school while those with university qualifications betting 15.3 fewer times than someone who did not complete high school. Employment status and income are uncorrelated with the number of non-sports bets. Respondents whose parents were both born overseas place on average 4.4 fewer bets than people whose parents were both born in Australia.

Again, focusing on results significant at the 1% level, results for the number of sports bets show that women place on average 18.2 fewer bets than men per year. In contrast to non-sports bets, the number of sports bets placed decreases with age; a person 10-years older bets on average 5.0 fewer times per year. The interaction between age and gender confirms that the gender effect on the number of sports bets does vary with age—supported relative to the model without the interactions, LR test = 37.52 (*p* value = 0.00). However, the marginal effect of gender on the number of sports bets decreases with age, with 18-year-old women placing 50 fewer sports bets than 18-year-old men. This difference is as low as 10 fewer sports bets per year when comparing women and men aged over 60 years: panel (b), Fig. 2. Education effects are similar to those for non-sports bets. People with trade qualifications bet on average 6.7 (10.0) times less than someone who did not complete high school. The only employment status category that is related to the number of sports bets is being a student, who place on average 10.3 fewer bets than the self-employed. The number of sports bets is related to income with most annual income categories above $40,000 betting on average between 8 and 11 more times per year, though some differences are significant only at the 5% level and others are smaller with 5 more bets per year and significant at the 10% level. Finally, respondents whose parents were both born overseas place 4.2 fewer sport bets on average than people whose parents were both born in Australia.

### Attitudes to Sports Betting

Selected results of estimating the model presented in Eq. (5) using the responses to the first set of questions on general attitudes about sports betting summarized in Table 2 are presented in Tables 5 and 6. Full results of these models are available in Online Resource 1 and Online Resource 2 of the Supplementary Materials, where estimates for all variables included in the models are presented. In all these models, an interaction between gender and all age terms (up to third order age terms are included in all models) is considered with the result of a LR test of the restriction that the coefficients on the interactions are zero presented in the last row of each column. In cases where the restriction is rejected and the interaction is non-zero (*p* value < 0.05), the model presented includes the interactions.

**Table 5:** Selected results of general attitudes about gambling and sports betting with focus on type of gambling behaviour included as explanatory variables

Variables	(1)	(2)	(3)	(4)	(5)
*Type of gambling (base category is no betting)*					
Non-sports betting	− 0.195***	− 0.166***	− 0.164***	− 0.101***	0.069***
	(0.019)	(0.019)	(0.019)	(0.020)	(0.019)
Sports betting	− 0.712***	− 0.530***	− 0.437***	− 0.318***	0.269***
	(0.024)	(0.025)	(0.025)	(0.026)	(0.025)
Sports and non-sports betting	− 0.718***	− 0.536***	− 0.506***	− 0.370***	0.299***
	(0.024)	(0.025)	(0.025)	(0.026)	(0.025)
Female	0.073***	− 0.001	0.048***	− 0.072***	− 0.133***
	(0.018)	(0.018)	(0.018)	(0.019)	(0.018)
Age	0.006***	− 0.003***	− 0.001	− 0.000	0.001
	(0.001)	(0.001)	(0.001)	(0.001)	(0.001)
Regional	− 0.031*	− 0.069***	− 0.049***	− 0.096***	0.040**
	(0.018)	(0.019)	(0.019)	(0.019)	(0.019)
*Highest education (base category—less than high school)*					
Completed high school	0.029	0.008	− 0.014	− 0.022	− 0.036
	(0.030)	(0.031)	(0.031)	(0.031)	(0.031)
TAFE or trade certificate	0.114***	0.077***	0.068**	0.067**	− 0.097***
	(0.028)	(0.028)	(0.028)	(0.029)	(0.029)
University	0.188***	0.148***	0.128***	0.163***	− 0.203***
	(0.025)	(0.026)	(0.026)	(0.027)	(0.026)
Observations	14,813	14,813	14,813	14,813	14,813
*R*-squared	0.121	0.057	0.047	0.032	0.033
*LR* test of age gender interaction (*p* value)	0.341	0.132	0.003	0.009	0.277

Dependant variable modelled in each column is response to the respectively labelled statement below. Responses to each question are standardised to have a mean of zero and standard deviation of one. Age is included as a third order polynomial with average marginal effects presented. *P* value of likelihood ratio (*LR*) test of interaction between age and gender presented in last row with interaction included if *p* value < 0.05. Standard errors in parentheses. Level of statistical significance denoted by *(10%), **(5%) and ***(1%). All models also include controls for marital status, being a parent, employment status, income bands, country of birth, parent’s country of birth, ATSI and health condition or disability over past 6 months – full results available in Supplementary Materials

(1): Sports betting should not be part of experiencing sport

(2): People who bet regularly on sport are at risk of harm from gambling

(3): Sports betting can place people at higher risk of relationship problems, mental health and wellbeing issues and money worries

(4): Regular discussion of the ‘odds’ when talking about sport can lead to gambling problems in individuals

(5): It’s easy for people with sports betting issues to stop gambling

**Table 6:** Selected results of general attitudes about gambling and sports betting with focus on the number of non-sport and sport bets per year as explanatory variables

Variables	(1)	(2)	(3)	(4)	(5)
Number non-sports bets/year	− 0.144***	− 0.138***	− 0.130***	− 0.089***	0.067***
(100’s)	(0.014)	(0.014)	(0.014)	(0.014)	(0.014)
Number sports bets/year	− 0.298***	− 0.262***	− 0.236***	− 0.152***	0.159***
(100’s)	(0.017)	(0.017)	(0.017)	(0.017)	(0.017)
Female	0.109***	0.018	0.058***	− 0.059***	− 0.140***
	(0.018)	(0.019)	(0.019)	(0.019)	(0.019)
Age	0.009***	− 0.000	0.001	0.001	− 0.000
	(0.001)	(0.001)	(0.001)	(0.001)	(0.001)
Regional	− 0.024	− 0.068***	− 0.042**	− 0.090***	0.029
	(0.019)	(0.019)	(0.019)	(0.019)	(0.019)
*Highest education (base category—less than high school)*					
Completed high school	0.015	− 0.001	− 0.025	− 0.033	− 0.024
	(0.031)	(0.032)	(0.032)	(0.032)	(0.032)
TAFE or trade certificate	0.107***	0.070**	0.054*	0.058*	− 0.094***
	(0.029)	(0.029)	(0.029)	(0.030)	(0.029)
University	0.207***	0.161***	0.132***	0.169***	− 0.203***
	(0.026)	(0.027)	(0.027)	(0.027)	(0.027)
Observations	14,293	14,293	14,293	14,293	14,293
*R*-squared	0.069	0.037	0.032	0.023	0.027
*LR* test of age gender interaction (*p* value)	0.152	0.879	0.082	0.025	0.385

Dependant variable modelled in each column is response to the respectively labelled statement below. Responses to each question are standardised to have a mean of zero and standard deviation of one. Age is included as a third order polynomial with average marginal effects presented. *P*-value of likelihood ratio (*LR*) test of interaction between age and gender presented in last row with interaction included if *p*-value < 0.05. Standard errors in parentheses. Level of statistical significance denoted by *(10%), **(5%) and ***(1%). All models also include controls for marital status, being a parent, employment status, income bands, country of birth, parent’s country of birth, ATSI and health condition or disability over past 6 months—full results available in Supplementary Materials

(1): Sports betting should not be part of experiencing sport

(2): People who bet regularly on sport are at risk of harm from gambling

(3): Sports betting can place people at higher risk of relationship problems, mental health and wellbeing issues and money worries

(4): Regular discussion of the ‘odds’ when talking about sport can lead to gambling problems in individuals

(5): It’s easy for people with sports betting issues to stop gambling

Results in Table 5 are for the model estimated with all the demographic control variables in ${X}_{i}$ together with a set of indicators for each type of betting behaviour, ${G}_{i}$, including non-sports betting, sports betting and both sports and non-sports betting, with no betting the omitted base category. Each column presents estimates for a model of standardized responses (mean zero and standard deviation of one) to a separate statement about sports betting. The statements upon which each dependent variable is based are listed in the table notes. Focusing on results significant at the 1% level (denoted by ***), the results show that after controlling for a large set of individual demographic characteristics, people who bet are on average less concerned about sport betting issues than non-bettors. This is evident for all 5 statements modelled. We can see in column (1), for example, responses to the statement ‘sports betting should not be part of experiencing sport’, relative to non-bettors, the average response of people who bet on non-sport only is 0.20 standard deviations lower, while people who bet on sport (only or both sport and non-sport) have an average response that is 0.71 standard deviations lower. Other key results from these models are that females are more concerned about sports betting than males, except for in their responses to the statement ‘people who bet regularly on sport are at risk of harm from gambling’, where there is no difference between men and women. The relationship with age is statistically significant for the statements ‘sports betting should not be part of experiencing sport’ and ‘people who bet regularly on sport are at risk of harm from gambling’ but the effects are small, with a 10-year older person having on average a 0.06 standard deviation higher response to the former question and a 0.03 standard deviation lower response to the latter. People from regional locations are on average more concerned about sports betting than people from metropolitan locations; however, this concern is not evident in response to ‘sports betting should not be part of experiencing sport’. The results on education show that relative to the base case of ‘did not complete high school’, those with trade qualifications are on average more concerned about sports betting and in turn, people with university education are even more concerned with even greater differences evident than for those with trade qualifications.

The results presented in Table 6 are for models of the same questions with the same demographic controls included but with betting behaviour replaced by the number of sports and non-sports bets, ${B}_{i}/100.$ Once again, each column presents estimates for a model of standardized responses (mean zero and standard deviation of one) to a separate statement about sports betting. The statements upon which each dependent variable is based are listed in the notes to the table. The impacts of the demographic characteristics in Table 6 are qualitatively similar to those found in Table 5. The key difference between Tables 5 and 6 is that gambling categories are replaced with the number of sports bets and the number of non-sports bets. On average, people who bet more often are less concerned about sports betting. However, 100 more sports bets per year (approximately 2 bets per week) has nearly double the impact on responses of 100 more non-sports bets. For example, responses to ‘sports betting should not be part of experiencing sport’ are on average 0.14 standard deviations lower for every additional 100 non-sports bets but are 0.30 standard deviations lower for every additional 100 sports bets.

The above analysis is repeated for the second set of statements summarized in Table 2 which focus on perceptions of the sports betting attitudes and behaviours of others. Selected results of this analysis with betting categories included are presented in Table 7 and with the number of bets included are presented in Table 8. Each column presents estimates for a model of responses to a separate statement about sports betting. Survey responses used to estimate each model have been standardized to have mean zero and standard deviation of one. The statements upon which each dependent variable is based are listed in the notes to each table. Full results of these models are available in Online Resource 3 and Online Resource 4 of the Supplementary Materials.

**Table 7:** Selected results of perceptions of other’s attitudes and behaviours with respect to sports betting with focus on type of gambling behaviour included as explanatory variables

Variables	(1)	(2)	(3)	(4)	(5)	(6)	(7)
*Type of gambling (base category is no betting)*							
Non-sports betting	− 0.032*	− 0.056***	0.177***	0.074***	0.155***	0.111***	0.053***
	(0.019)	(0.019)	(0.019)	(0.019)	(0.018)	(0.017)	(0.018)
Sports betting	− 0.022	0.158***	0.561***	0.650***	0.497***	0.720***	0.490***
	(0.025)	(0.025)	(0.025)	(0.025)	(0.024)	(0.022)	(0.023)
Sports and non-sports betting	− 0.003	0.085***	0.615***	0.654***	0.506***	0.686***	0.483***
	(0.025)	(0.025)	(0.025)	(0.025)	(0.024)	(0.022)	(0.023)
Female	0.117***	0.030*	0.103***	0.186***	− 0.172***	− 0.385***	− 0.418***
	(0.019)	(0.018)	(0.018)	(0.018)	(0.018)	(0.016)	(0.017)
Age	− 0.009***	− 0.013***	− 0.005***	− 0.004***	− 0.014***	− 0.017***	− 0.016***
	(0.001)	(0.001)	(0.001)	(0.001)	(0.001)	(0.001)	(0.001)
Regional	− 0.009	− 0.018	0.008	− 0.010	0.030*	0.003	− 0.062***
	(0.019)	(0.018)	(0.018)	(0.018)	(0.018)	(0.017)	(0.017)
*Highest education (base category—less than high school)*							
Completed high school	− 0.065**	− 0.110***	− 0.085***	− 0.114***	− 0.062**	− 0.095***	− 0.083***
	(0.031)	(0.030)	(0.030)	(0.030)	(0.030)	(0.028)	(0.029)
TAFE or trade certificate	− 0.071**	− 0.093***	− 0.141***	− 0.125***	− 0.134***	− 0.112***	− 0.115***
	(0.029)	(0.028)	(0.028)	(0.028)	(0.027)	(0.025)	(0.027)
University	− 0.154***	− 0.219***	− 0.220***	− 0.194***	− 0.205***	− 0.218***	− 0.134***
	(0.026)	(0.026)	(0.026)	(0.025)	(0.025)	(0.023)	(0.024)
Observations	14,813	14,813	14,813	14,813	14,813	14,813	14,813
*R*-squared	0.043	0.075	0.089	0.110	0.143	0.262	0.194
*LR* test of age gender interaction (*p*-value)	0.004	0.000	0.000	0.000	0.965	0.000	0.000

Dependant variable modelled in each column is response to the respectively labelled statement below. Responses to each question are standardised to have a mean of zero and standard deviation of one. Age is included as a third order polynomial with average marginal effects presented. *P* value of likelihood ratio (*LR*) test of interaction between age and gender presented in last row with interaction included if *p* value < 0.05. Standard errors in parentheses. Level of statistical significance denoted by *(10%), **(5%) and ***(1%). All models also include controls for marital status, being a parent, employment status, income bands, country of birth, parent’s country of birth, ATSI and health condition or disability over past 6 months—full results available in Supplementary Materials

(1): Most people in society think betting on sport is harmless

(2): Most people in society bet on sport

(3): Most people in my family think betting on sport is harmless

(4): Most people in my family bet on sport

(5): Most people in my friendship group think betting on sport is harmless

(6): Most people in my friendship group bet on sport

(7): Odds talk is common in discussions about sport with my friends and peers

**Table 8:** Selected results of models of perceptions of other’s attitudes and behaviours with respect to sports betting with focus on the number of non-sport and sport bets per year as explanatory variables

Variables	(1)	(2)	(3)	(4)	(5)	(6)	(7)
Number non-sports bets/year	− 0.008	− 0.005	0.111***	0.083***	0.106***	0.102***	0.088***
(100’s)	(0.014)	(0.014)	(0.014)	(0.014)	(0.014)	(0.013)	(0.013)
Number sports bets/year	0.016	0.121***	0.216***	0.335***	0.203***	0.375***	0.300***
(100’s)	(0.017)	(0.017)	(0.017)	(0.017)	(0.017)	(0.016)	(0.016)
Female	0.120***	0.017	0.066***	0.141***	− 0.203***	− 0.423***	− 0.441***
	(0.019)	(0.018)	(0.019)	(0.018)	(0.018)	(0.017)	(0.017)
Age	− 0.009***	− 0.013***	− 0.008***	− 0.007***	− 0.016***	− 0.020***	− 0.018***
	(0.001)	(0.001)	(0.001)	(0.001)	(0.001)	(0.001)	(0.001)
Regional	− 0.015	− 0.018	− 0.000	− 0.016	0.025	− 0.002	0.064***
	(0.019)	(0.019)	(0.019)	(0.019)	(0.019)	(0.017)	(0.018)
*Highest education (base category—less than high school)*							
Completed high school	− 0.054*	− 0.099***	− 0.076**	− 0.108***	− 0.057*	− 0.090***	− 0.079***
	(0.032)	(0.031)	(0.032)	(0.031)	(0.031)	(0.029)	(0.030)
TAFE or trade certificate	− 0.062**	− 0.094***	− 0.136***	− 0.114***	− 0.132***	− 0.100***	− 0.103***
	(0.029)	(0.029)	(0.029)	(0.029)	(0.028)	(0.027)	(0.027)
University	− 0.138***	− 0.215***	− 0.243***	− 0.205***	− 0.228***	− 0.237***	− 0.141***
	(0.027)	(0.026)	(0.027)	(0.026)	(0.026)	(0.024)	(0.025)
Observations	14,293	14,293	14,293	14,294	14,293	14,293	14,293
*R*-squared	0.043	0.075	0.053	0.067	0.118	0.215	0.176
*LR* test of age gender interaction (*p*-value)	0.006	0.001	0.004	0.006	0.285	0.000	0.000

Dependant variable modelled in each column is response to the respectively labelled statement below. Responses to each question are standardised to have a mean of zero and standard deviation of one. *P* value of likelihood ratio (*LR*) test of interaction between age and gender presented in last row with interaction included if *p* value < 0.05. Standard errors in parentheses. Level of statistical significance denoted by *(10%), **(5%) and ***(1%). All models also include controls for marital status, being a parent, employment status, income bands, Country of birth, parent’s country of birth, ATSI and health condition or disability over past 6 months—full results available in Supplementary Materials

(1): Most people in society think betting on sport is harmless

(2): Most people in society bet on sport

(3): Most people in my family think betting on sport is harmless

(4): Most people in my family bet on sport

(5): Most people in my friendship group think betting on sport is harmless

(6): Most people in my friendship group bet on sport

(7): Odds talk is common in discussions about sport with my friends and peers

**Fig. 3:**
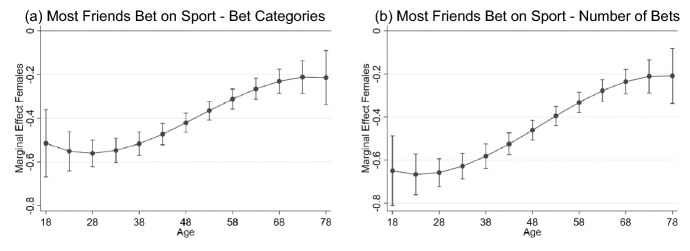
Marginal effect of gender on response to the question “Most people in my friendship group bet on sport”, plotted over age. Panel (**a**) is based on model in column (6) from Table 7 which includes betting categories and panel (**b**) is based on model in column (6) from Table 8 which includes number of bets. Point estimates are denoted by dots with 95% confidence intervals for each point estimate included

Focusing on significance at the 1% level (denoted by ***), Table 7 shows, except for the first two statements that focus on attitudes in society, bettors have families and friendship groups where gambling is common and perceived as harmless. However, the difference between sports bettors and non-sports bettors is stark. For example, in response to the question ‘most people in my friendship group bet on sport’, the average response of non-sports bettors is 0.16 standard deviations higher than non-bettors whereas the average response of sports bettors is up to 0.50 standard deviations higher, with both significant at 1%. The difference between non-bettors and sports bettors (0.49 standard deviations) is nearly 10 times as large as the difference between non-bettors and non-sports bettors (0.05 standard deviations) in response to the question ‘odds talk is common in discussions about sport with my friends and peers’. The largest impact of age is on the peer and friendship group statements, columns (5)–(7), with a response of a person 10-years younger on average 0.15 standard deviations higher. The interaction between gender and age is illustrated for the results reported in column (6) which is based on the statement “most people in my friendship group bet on sport” in panel (a) of Fig. 3. The difference between men and women is greatest at younger ages with women in the 18–45 year range responding on average 0.5 standard deviations lower than men, suggesting young men have a much stronger belief than young women that their friends are involved in sports betting.

The results presented in Table 8 are for models of the same questions analyzed in Table 7, with the same demographic controls included but with betting behaviour replaced by the number of sports and non-sports bets, ${B}_{i}/100.$ The relationships with the demographic characteristics are qualitatively similar to the results presented in Table 7 and discussed above. The more a respondent bets, the greater their agreement with all but the first two statements that focus on attitudes in society. The relationship between responses and number of sports bets is up to 4 times as large as the relationship with the number of non-sports bets. For example, responses to the question ‘most people in my friendship group bet on sport’ on average increase by 0.10 standard deviations for people who place 100 more non-sports bets per year (2 more bets per week) but for an otherwise identical person who places an additional 100 sports bets, their response is on average 0.38 standard deviations higher. These sorts of differences are evident across all questions about family and friendship groups, with a greater number of bets associated with responses that show sports betting is believed to be more common and perceived as less harmful in these circles. It is also found the more sports bets a person places, the stronger their agreement with the statement ‘most people in society bet on sport’, though the relationship with the number of non-sports bets is statistically insignificant. The gender and age interaction for the model in column (6) in Table 8 is presented in panel (b) of Fig. 3. The figure shows the difference between men and women is greatest among 18–28-year-olds, with the responses of women in this age range on average 0.70 standard deviations lower. This compares with differences of less than 0.30 standard deviations for those over 60 years. The results are consistent with those in panel (a) of Fig. 3, suggesting that gender age differences are robust whether we control for the betting category or the number of bets per year.

## Discussion

This study builds on existing literature at the intersection of sports betting and sports, providing a comprehensive analysis of the sports betting behaviour of sports fans, including many people who choose not to gamble at all. Survey respondents’ attitudes to sports betting were analysed using betting behaviour and a wide range of demographic characteristics. The approach differs from many previous studies as we targeted a broader demographic of sports fans, rather than focusing only on those engaged in gambling (sports or non-sports), which is a strength. We did not measure whether a person’s gambling behaviour is deemed ‘problematic’, but previous research has demonstrated a connection between frequency of sports betting and problematic gambling behaviour (Hing et al., 2016). Therefore, our analysis of the number of sports bets provides a useful proxy to identify those most at risk of experiencing gambling harm. The research was guided by two overarching questions addressed in turn through the following discussion.

### Demographic Profile of Sports Bettors

The dominant theme emerging from our analysis is the importance of gender, age and their interaction. The gender difference in the probability of sports betting is wider among the youngest in the sample; 18-year-old men are about 25 percentage points more likely than their female counterparts to bet on sports, whereas this difference is less than 5 percentage points for those over 60 years. Similar patterns are evident for the number of sports bets placed, with younger men placing more bets than similar aged women and fewer bets being placed with each additional year of age. Consequently, young men are most at risk based on their sports betting engagement and number of bets placed. This aligns with previous studies (Hing et al., 2016; Williams et al., 2012), but widens our understanding of the sports betting behaviour of sports fans. Moreover, even though this has been described in smaller-scale qualitative research studies (Deans et al., 2017b; Waitt et al., 2020) our results are based on empirical analytic techniques applied to a larger and more diverse sample. The results comprehensively demonstrate sports betting is predominantly pursued by young men, in sharp contrast to other forms of gambling. Given the recent growth of sports betting, its marketing, and increasing contribution to problem gambling (Hing et al., 2019), as well as the need for appropriately tailored prevention and early intervention public health initiatives, this finding is significant for highlighting the distinctive sports betting behaviour of young men aged 18–35. Recent research has started to examine young women aged 18–35 as an emerging gambling cohort (see McCarthy et al., 2020), but our results demonstrate no significant gender or age effects for women’s sports and non-sports betting behaviour.

Other important demographic factors included education level, relationship status, and employment status. People who are widowed or separated were less likely to bet on sports, but no other relationship types were significant at the 1% level. University educated individuals were less likely to bet on sports than those who did not complete high school. Employment status did not exhibit a strong relationship with sports betting, except students and unemployed were less likely than self-employed to bet on sports. Surprisingly, income did seemingly not influence whether people engaged in sports betting only. This was more important in the context of making both sports and non-sports bets—those reporting higher levels of income were more likely to engage in these gambling types.

Whilst other studies have reported various demographic risk factors for sports betting and gambling, our results contribute by clearly demonstrating the significant interaction between age and gender. The importance of our study for public health policy and harm reduction campaign strategies is twofold. First, our sampling frame is likely the target audience of sports betting marketers, providing strong evidence upon which to base public health policy and harm reduction campaigns. Second, such campaigns should be aimed specifically at young men to help counteract the increasing environmental and social normalisation of sports betting. The next section focuses on the key attitudinal differences that emerged from the results to answer our second research question.

### Attitudes Associated with Sports Betting

Our results demonstrate there are significant differences between the attitudes of sports bettors (either sports betting or sports betting combined with non-sports betting), non-sport bettors and non-bettors. Not only do sports bettors feel more strongly that sports betting has a place in sport, they are also less concerned about the risks and harms of sports betting. These results help to demonstrate the effects of the normalisation processes outlined in previous studies. Whilst existing literature has documented how sports and sports betting have become synonymous (Milner et al., 2013; Nyemcosk et al., 2021; Pitt et al., 2016a; Thomas, 2018), the attitudinal differences we identified highlight how the ‘gamblification’ (McGee, 2020) of sports has penetrated individual perceptions about sports betting as an activity and influenced behaviour. Moreover, the differences between sports bettors and non-sports bettors suggest something unique is happening for this group; it is not necessarily related to the act of gambling, but potentially broader environmental and socio-cultural influences. Gender and age effects are also apparent, with women less likely to agree that sports betting should be part of experiencing sports and more likely to agree that sports betting can place people at higher risk of other harms. A similar pattern is evident for age, with younger people generally being more permissive of sports betting.

The influence of the social aspects of sports betting, namely the characteristics of sports bettors’ social networks, is also a strong emerging theme, underpinned by several attitudinal measures. Sports bettors are more likely to have family and friendship groups where gambling is common and perceived as relatively harmless. Additionally, they are more likely to agree that discussions about odds and the placing of bets is the norm amongst peers. In this context, there are significant differences observed between both sports bettors and non-bettors, and sports bettors and non-sports bettors. This again highlights that there are potentially distinct socialisation processes specifically influencing the attitudes and behaviours of sports bettors. Whilst this has previously been described on a relatively smaller scale (Thomas, 2017a), our research demonstrates that these interactional and socialisation factors are highly meaningful in an extensive cohort of sports fans.

Age and gender are the key demographic factors related to responses in a similar way to that described in the previous section. Whilst women are more likely to agree that sports betting is common in broader society and amongst family members, men are more likely to indicate it is common within their peer groups. Men are also more likely to state that ‘odds talk’ is prevalent when socialising with their peers. In combination with the demographic risk factors outlined in the previous section, it is apparent that men also have different interactions with sports betting. They are more likely to agree it has a place in sports, less likely to think it is risky and can lead to other harms, more likely to have friends and peers who bet on sports, and more likely to have dialogue that supports and endorses the normalisation of sports betting. These attitudes combined suggest it is imperative public health prevention measures and harm reduction interventions target young men. The impact of peer socialisation processes and hegemonic masculine norms around sports betting have been described in previous studies (Ayandele et al., 2019; Bunn et al., 2019; Deans et al., 2017a). Sports betting has also been related to the development of socially valorised identities for young men (Lamont and Hing, 2021). Our research supports and builds on these previous findings by demonstrating in a large sample that it has become a more prevalent part of sports fandom for younger adult men.

## Conclusion

On a large and unique scale, we have demonstrated fundamental attitudinal and behavioural differences, and distinct and concerning trends, among those who engage in sports betting, thereby offering important insights about those most at risk. Most previous research has been qualitative or focused on those identified as having problematic gambling behaviours. By contrast, the scale and type of results generated from this study have afforded the ability to compare differences between non-bettors and bettors, providing compelling evidence of current issues amongst a general cohort of sports fans. Importantly, this study provides data about an emerging public health crisis in which younger men are most at risk because they are more exposed to sports betting normalisation processes, show greater engagement with sports betting and express more permissive attitudes. As such, the results of this study provide a foundation for public health interventions and programs.

## Supplementary Information

Below is the link to the electronic supplementary material.

Supplementary file1 (DOCX 90 KB)

## Footnotes

1 One participant reported 52,000 bets per year while some others reported 5,200 and 2,600 bets per year. Such observations were treated as outliers and excluded from the analysis. This amounted to 14 observations being excluded from the analysis.

2 Higher order age terms are not supported by a likelihood ratio (LR) test of a model with a fourth order age term with a test statistic of 4.63 (*p*-value = 0.20).

3 Higher order age terms are not supported by a likelihood ratio (LR) test of a model with a fourth order age term with a test statistic of 3.33 (*p*-value = 0.19) for the model of the number of non-sports bets and 2.74 (*p*-value = 0.25) for the model of the number of sports bets.
