# Supplementary material for: The Gambling Behaviour and Attitudes to Sports Betting of Sports Fans
Source: J Gambl Stud. 2022 Feb 1;38(4):1371–403. doi: 10.1007/s10899-021-10101-7 (PMC8806135; doi:10.1007/s10899-021-10101-7)
Supplement: Supplementary file 1 — Supplementary file1 (DOCX 90 KB) [file 10899_2021_10101_MOESM1_ESM.docx]

# Supplementary Materials – Online Resource 1

This online resource provides tables of full results for models presented in Table 5, 6, 7 and 8 of the paper “The Gambling Behaviour and Attitudes to Sports Betting of Sports Fans” where selected results are given.

| Table 1: Full results of estimated models of general attitudes about gambling and sports betting with focus on type of gambling behaviour included as explanatory variables. Table corresponds to Table 5 of paper. | | | | | |
| --- | --- | --- | --- | --- | --- |
| Variables | (1) | (2) | (3) | (4) | (5) |
| **Type of gambling (base category is no betting)** | | |  |  |  |
| Non-sport betting | -0.195*** | -0.166*** | -0.164*** | -0.101*** | 0.069*** |
|  | (0.019) | (0.019) | (0.019) | (0.020) | (0.019) |
| Sport betting | -0.712*** | -0.530*** | -0.437*** | -0.318*** | 0.269*** |
|  | (0.024) | (0.025) | (0.025) | (0.026) | (0.025) |
| Sport and non-sport | -0.718*** | -0.536*** | -0.506*** | -0.370*** | 0.299*** |
|  | (0.024) | (0.025) | (0.025) | (0.026) | (0.025) |
| Female | 0.073*** | -0.001 | 0.048*** | -0.072*** | -0.133*** |
|  | (0.018) | (0.018) | (0.018) | (0.019) | (0.018) |
| Age | 0.006*** | -0.003*** | -0.001 | -0.000 | 0.001 |
|  | (0.001) | (0.001) | (0.001) | (0.001) | (0.001) |
| Regional | -0.031* | -0.069*** | -0.049*** | -0.096*** | 0.040** |
|  | (0.018) | (0.019) | (0.019) | (0.019) | (0.019) |
| **Marital Status** |  |  |  |  |  |
| Defactor (live together) | 0.029 | -0.025 | 0.038 | 0.025 | -0.122*** |
|  | (0.030) | (0.031) | (0.031) | (0.031) | (0.031) |
| Defactor (live apart) | -0.019 | -0.019 | 0.011 | 0.002 | -0.038 |
|  | (0.047) | (0.048) | (0.048) | (0.049) | (0.048) |
| Married/Civil Union | 0.015 | -0.028 | 0.001 | -0.011 | -0.060** |
|  | (0.026) | (0.027) | (0.027) | (0.028) | (0.027) |
| Separated/Divorced | 0.009 | -0.015 | -0.009 | -0.011 | -0.069 |
|  | (0.041) | (0.043) | (0.043) | (0.044) | (0.043) |
| Widowed | -0.125** | -0.169*** | -0.110* | -0.075 | -0.118* |
|  | (0.060) | (0.062) | (0.062) | (0.064) | (0.062) |
| Parent | 0.021 | 0.018 | 0.012 | -0.016 | 0.001 |
|  | (0.020) | (0.020) | (0.020) | (0.021) | (0.020) |
| **Highest education (base category – less than high school)** | | | | | |
| Completed high school | 0.029 | 0.008 | -0.014 | -0.022 | -0.036 |
|  | (0.030) | (0.031) | (0.031) | (0.031) | (0.031) |
| TAFE or trade certificate | 0.114*** | 0.077*** | 0.068** | 0.067** | -0.097*** |
|  | (0.028) | (0.028) | (0.028) | (0.029) | (0.029) |
| University | 0.188*** | 0.148*** | 0.128*** | 0.163*** | -0.203*** |
|  | (0.025) | (0.026) | (0.026) | (0.027) | (0.026) |
| **Employment (base - self employed)** | |  |  |  |  |
| Employed (wage/salary) | 0.016 | 0.017 | 0.029 | -0.062** | -0.010 |
|  | (0.024) | (0.025) | (0.025) | (0.025) | (0.025) |
| Unemployed | 0.003 | 0.030 | 0.016 | -0.065 | 0.013 |
|  | (0.058) | (0.059) | (0.059) | (0.060) | (0.060) |
| Home duties | 0.082 | -0.005 | 0.052 | 0.060 | -0.030 |
|  | (0.069) | (0.072) | (0.072) | (0.073) | (0.072) |
| Student | 0.147** | 0.046 | 0.090 | -0.054 | -0.039 |
|  | (0.064) | (0.066) | (0.066) | (0.067) | (0.066) |
| Retired | 0.038 | 0.018 | 0.019 | -0.002 | -0.026 |
|  | (0.033) | (0.034) | (0.034) | (0.035) | (0.034) |
| Unable to work | -0.054 | -0.021 | 0.073 | -0.047 | 0.025 |
|  | (0.084) | (0.086) | (0.086) | (0.088) | (0.087) |
| **Income (base - less than $10,000)** | |  |  |  |  |
| $10k- less than $20k | 0.008 | -0.028 | 0.103 | -0.008 | -0.242** |
|  | (0.098) | (0.101) | (0.101) | (0.103) | (0.102) |
| $20k - less than $30k | 0.023 | 0.150* | 0.261*** | 0.166* | -0.315*** |
|  | (0.087) | (0.090) | (0.090) | (0.092) | (0.090) |
| $30k - less than $40k | 0.011 | 0.076 | 0.182** | 0.128 | -0.201** |
|  | (0.087) | (0.089) | (0.089) | (0.091) | (0.090) |
| $40k - less than $50k | 0.008 | 0.043 | 0.034 | 0.212** | -0.183** |
|  | (0.085) | (0.087) | (0.087) | (0.089) | (0.088) |
| $50k - less than $60k | 0.063 | 0.080 | 0.154* | 0.189** | -0.150* |
|  | (0.083) | (0.085) | (0.085) | (0.087) | (0.086) |
| $60k - less than $80k | -0.010 | 0.068 | 0.172** | 0.167** | -0.274*** |
|  | (0.079) | (0.082) | (0.082) | (0.083) | (0.082) |
| $80k - less than $100k | 0.011 | 0.090 | 0.170** | 0.178** | -0.258*** |
|  | (0.079) | (0.082) | (0.082) | (0.083) | (0.082) |
| $100k - less than $125k | 0.024 | 0.065 | 0.155* | 0.150* | -0.271*** |
|  | (0.080) | (0.082) | (0.082) | (0.083) | (0.082) |
| $1250k - less than $150k | -0.012 | 0.088 | 0.133 | 0.157* | -0.295*** |
|  | (0.081) | (0.083) | (0.083) | (0.084) | (0.083) |
| $150k - less than $200k | -0.000 | 0.030 | 0.162** | 0.167** | -0.255*** |
|  | (0.080) | (0.082) | (0.082) | (0.084) | (0.083) |
| $200k or more | 0.042 | 0.069 | 0.177** | 0.202** | -0.311*** |
|  | (0.080) | (0.083) | (0.083) | (0.084) | (0.083) |
| Prefer not to say | 0.037 | 0.051 | 0.131* | 0.167** | -0.221*** |
|  | (0.077) | (0.080) | (0.080) | (0.081) | (0.080) |
| **Country of birth (base - Australia)** | |  |  |  |  |
| UK or New Zealand | 0.001 | -0.032 | -0.030 | -0.061 | -0.145*** |
|  | (0.038) | (0.039) | (0.039) | (0.040) | (0.039) |
| Other | -0.065 | 0.193** | 0.180* | 0.039 | -0.096 |
|  | (0.091) | (0.094) | (0.094) | (0.095) | (0.094) |
| **Parent's Country of birth (base - both Australia)** | | |  |  |  |
| One in Australia | 0.010 | 0.033 | 0.043* | 0.015 | 0.010 |
|  | (0.023) | (0.023) | (0.023) | (0.024) | (0.024) |
| Both Overseas | 0.042* | 0.045* | 0.060** | -0.008 | 0.084*** |
|  | (0.024) | (0.025) | (0.025) | (0.025) | (0.025) |
| ATSI | -0.013 | -0.075 | 0.050 | 0.075 | 0.065 |
|  | (0.086) | (0.088) | (0.088) | (0.090) | (0.089) |
| Disability or health issue (> 6 months) | 0.024 | 0.031 | -0.002 | 0.011 | -0.010 |
|  | (0.027) | (0.027) | (0.027) | (0.028) | (0.027) |
| Constant | -0.072 | 0.821*** | 0.198 | -0.516* | 0.178 |
|  | (0.238) | (0.245) | (0.245) | (0.291) | (0.246) |
| Observations | 14,813 | 14,813 | 14,813 | 14,813 | 14,813 |
| R-squared | 0.121 | 0.057 | 0.047 | 0.032 | 0.033 |
| LR test of age gender interaction (*p*-value) | 0.341 | 0.132 | 0.003 | 0.009 | 0.277 |
| Notes: Dependant variable modelled in each column is response to the respectively labelled statement below. Responses to each question are standardised to have a mean of zero and standard deviation of one. Age is included as a third order polynomial with average marginal effects presented. P-value of likelihood ratio (LR) test of interaction between age and gender presented in last row with interaction included if *p*-value < 0.05. Standard errors in parentheses. Level of statistical significance denoted by * (10%), ** (5%) and *** (1%).  (1): Sports betting should not be part of experiencing sport.  (2): People who bet regularly on sport are at risk of harm from gambling.  (3): Sports betting can place people at higher risk of relationship problems, mental health and wellbeing issues and money worries.  (4): Regular discussion of the ‘odds’ when talking about sport can lead to gambling problems in individuals.  (5): It’s easy for people with sports betting issues to stop gambling. | | | | | |

| Table 2: Full results of estimated models of general attitudes about gambling and sports betting with focus on the number of non-sport and sport bets per year as explanatory variables. Table corresponds to Table 6 of paper. | | | | | |
| --- | --- | --- | --- | --- | --- |
| Variables | (1) | (2) | (3) | (4) | (5) |
| Number non-sport bets/year | -0.144*** | -0.138*** | -0.130*** | -0.089*** | 0.067*** |
| (100’s) | (0.014) | (0.014) | (0.014) | (0.014) | (0.014) |
| Number sport bets/year | -0.298*** | -0.262*** | -0.236*** | -0.152*** | 0.159*** |
| (100’s) | (0.017) | (0.017) | (0.017) | (0.017) | (0.017) |
| Female | 0.109*** | 0.018 | 0.058*** | -0.059*** | -0.140*** |
|  | (0.018) | (0.019) | (0.019) | (0.019) | (0.019) |
| Age | 0.009*** | -0.000 | 0.001 | 0.001 | -0.000 |
|  | (0.001) | (0.001) | (0.001) | (0.001) | (0.001) |
| Regional | -0.024 | -0.068*** | -0.042** | -0.090*** | 0.029 |
|  | (0.019) | (0.019) | (0.019) | (0.019) | (0.019) |
| **Marital Status** |  |  |  |  |  |
| Defacto (live together) | 0.011 | -0.040 | 0.023 | 0.017 | -0.110*** |
|  | (0.031) | (0.032) | (0.032) | (0.032) | (0.032) |
| Defacto (live apart) | -0.054 | -0.052 | -0.018 | -0.018 | -0.024 |
|  | (0.049) | (0.050) | (0.050) | (0.051) | (0.050) |
| Married/Civil Union | 0.009 | -0.046* | -0.019 | -0.021 | -0.047* |
|  | (0.027) | (0.028) | (0.028) | (0.028) | (0.028) |
| Separated/Divorced | -0.007 | -0.033 | -0.029 | -0.023 | -0.063 |
|  | (0.043) | (0.044) | (0.044) | (0.045) | (0.044) |
| Widowed | -0.121* | -0.175*** | -0.116* | -0.065 | -0.111* |
|  | (0.062) | (0.063) | (0.063) | (0.065) | (0.063) |
| Parent | 0.025 | 0.022 | 0.018 | -0.012 | 0.000 |
|  | (0.021) | (0.021) | (0.021) | (0.021) | (0.021) |
| **Highest education (base category – less than high school)** | | | |  |  |
| Completed high school | 0.015 | -0.001 | -0.025 | -0.033 | -0.024 |
|  | (0.031) | (0.032) | (0.032) | (0.032) | (0.032) |
| TAFE or trade certificate | 0.107*** | 0.070** | 0.054* | 0.058* | -0.094*** |
|  | (0.029) | (0.029) | (0.029) | (0.030) | (0.029) |
| University | 0.207*** | 0.161*** | 0.132*** | 0.169*** | -0.203*** |
|  | (0.026) | (0.027) | (0.027) | (0.027) | (0.027) |
| **Employment (base - self employed)** |  |  |  |  |  |
| Employed (wage/salary) | 0.014 | 0.015 | 0.024 | -0.067** | -0.007 |
|  | (0.025) | (0.026) | (0.025) | (0.026) | (0.025) |
| Unemployed | 0.031 | 0.062 | 0.073 | -0.062 | -0.016 |
|  | (0.061) | (0.062) | (0.061) | (0.062) | (0.061) |
| Home duties | 0.108 | 0.037 | 0.079 | 0.066 | -0.048 |
|  | (0.072) | (0.073) | (0.073) | (0.074) | (0.073) |
| Student | 0.199*** | 0.079 | 0.116* | -0.045 | -0.076 |
|  | (0.067) | (0.068) | (0.068) | (0.069) | (0.068) |
| Retired | 0.048 | 0.023 | 0.028 | 0.000 | -0.035 |
|  | (0.034) | (0.035) | (0.035) | (0.035) | (0.035) |
| Unable to work | -0.070 | -0.002 | 0.047 | -0.036 | 0.037 |
|  | (0.087) | (0.088) | (0.088) | (0.089) | (0.088) |
| **Income (base - less than $10,000)** |  |  |  |  |  |
| $10k- less than $20k | -0.025 | -0.110 | 0.039 | -0.073 | -0.221** |
|  | (0.103) | (0.105) | (0.104) | (0.106) | (0.104) |
| $20k - less than $30k | -0.026 | 0.085 | 0.228** | 0.128 | -0.329*** |
|  | (0.092) | (0.093) | (0.093) | (0.094) | (0.093) |
| $30k - less than $40k | -0.041 | -0.010 | 0.147 | 0.091 | -0.210** |
|  | (0.091) | (0.092) | (0.092) | (0.093) | (0.092) |
| $40k - less than $50k | -0.029 | -0.028 | -0.008 | 0.187** | -0.203** |
|  | (0.089) | (0.090) | (0.090) | (0.091) | (0.090) |
| $50k - less than $60k | 0.021 | 0.020 | 0.119 | 0.149* | -0.160* |
|  | (0.087) | (0.088) | (0.087) | (0.089) | (0.087) |
| $60k - less than $80k | -0.059 | 0.001 | 0.138 | 0.126 | -0.280*** |
|  | (0.083) | (0.085) | (0.084) | (0.086) | (0.084) |
| $80k - less than $100k | -0.057 | 0.006 | 0.108 | 0.131 | -0.261*** |
|  | (0.083) | (0.084) | (0.084) | (0.085) | (0.084) |
| $100k - less than $125k | -0.043 | -0.013 | 0.106 | 0.101 | -0.269*** |
|  | (0.084) | (0.085) | (0.084) | (0.086) | (0.084) |
| $1250k - less than $150k | -0.098 | -0.003 | 0.064 | 0.099 | -0.292*** |
|  | (0.084) | (0.086) | (0.085) | (0.087) | (0.085) |
| $150k - less than $200k | -0.071 | -0.055 | 0.110 | 0.120 | -0.261*** |
|  | (0.084) | (0.085) | (0.085) | (0.086) | (0.085) |
| $200k or more | -0.056 | -0.026 | 0.119 | 0.147* | -0.321*** |
|  | (0.084) | (0.085) | (0.085) | (0.086) | (0.085) |
| Prefer not to say | -0.018 | -0.020 | 0.086 | 0.127 | -0.233*** |
|  | (0.081) | (0.082) | (0.082) | (0.083) | (0.082) |
| **Country of birth (base - Australia)** |  |  |  |  |  |
| UK or New Zealand | -0.006 | -0.038 | -0.042 | -0.061 | -0.132*** |
|  | (0.040) | (0.040) | (0.040) | (0.041) | (0.040) |
| Other | -0.061 | 0.218** | 0.174* | 0.043 | -0.083 |
|  | (0.094) | (0.095) | (0.095) | (0.096) | (0.095) |
| **Parent's Country of birth (base - both Australia)** |  |  |  |  |  |
| One in Australia | 0.016 | 0.032 | 0.046* | 0.019 | 0.001 |
|  | (0.024) | (0.024) | (0.024) | (0.024) | (0.024) |
| Both Overseas | 0.058** | 0.053** | 0.076*** | 0.002 | 0.074*** |
|  | (0.025) | (0.026) | (0.026) | (0.026) | (0.026) |
| ATSI | 0.025 | -0.029 | 0.099 | 0.068 | -0.027 |
|  | (0.092) | (0.093) | (0.092) | (0.094) | (0.092) |
| Diability or health issue (> 6 months) | 0.042 | 0.032 | 0.011 | 0.012 | -0.001 |
|  | (0.028) | (0.028) | (0.028) | (0.028) | (0.028) |
| Constant | -0.158 | 0.782*** | 0.189 | -0.534* | 0.200 |
|  | (0.251) | (0.255) | (0.253) | (0.303) | (0.253) |
| Observations | 14,293 | 14,293 | 14,293 | 14,293 | 14,293 |
| R-squared | 0.069 | 0.037 | 0.032 | 0.023 | 0.027 |
| LR test of age gender interaction (*p*-value) | 0.152 | 0.879 | 0.082 | 0.025 | 0.385 |
| Notes: Dependant variable modelled in each column is response to the respectively labelled statement below. Responses to each question are standardised to have a mean of zero and standard deviation of one. Age is included as a third order polynomial with average marginal effects presented. P-value of likelihood ratio (LR) test of interaction between age and gender presented in last row with interaction included if *p*-value < 0.05. Standard errors in parentheses. Level of statistical significance denoted by * (10%), ** (5%) and *** (1%).  (1): Sports betting should not be part of experiencing sport.  (2): People who bet regularly on sport are at risk of harm from gambling.  (3): Sports betting can place people at higher risk of relationship problems, mental health and wellbeing issues and money worries.  (4): Regular discussion of the ‘odds’ when talking about sport can lead to gambling problems in individuals.  (5): It’s easy for people with sports betting issues to stop gambling. | | | | | |

| Table 3: Full results of estimated models of perceptions of other’s attitudes and behaviours with respect to sports betting, focusing on type of gambling included as explanatory variables. Table corresponds to Table 7 of paper. | | | | | | | |
| --- | --- | --- | --- | --- | --- | --- | --- |
| Variables | (1) | (2) | (3) | (4) | (5) | (6) | (7) |
| **Type of gambling (base category is no betting)** | | | |  |  |  |  |
| Non-sport betting | -0.032* | -0.056*** | 0.177*** | 0.074*** | 0.155*** | 0.111*** | 0.053*** |
|  | (0.019) | (0.019) | (0.019) | (0.019) | (0.018) | (0.017) | (0.018) |
| Sport betting | -0.022 | 0.158*** | 0.561*** | 0.650*** | 0.497*** | 0.720*** | 0.490*** |
|  | (0.025) | (0.025) | (0.025) | (0.025) | (0.024) | (0.022) | (0.023) |
| Sport and non-sport | -0.003 | 0.085*** | 0.615*** | 0.654*** | 0.506*** | 0.686*** | 0.483*** |
|  | (0.025) | (0.025) | (0.025) | (0.025) | (0.024) | (0.022) | (0.023) |
| Female | 0.117*** | 0.030* | 0.103*** | 0.186*** | -0.172*** | -0.385*** | -0.418*** |
|  | (0.019) | (0.018) | (0.018) | (0.018) | (0.018) | (0.016) | (0.017) |
| Age | -0.009*** | -0.013*** | -0.005*** | -0.004*** | -0.014*** | -0.017*** | -0.016*** |
|  | (0.001) | (0.001) | (0.001) | (0.001) | (0.001) | (0.001) | (0.001) |
| Regional | -0.009 | -0.018 | 0.008 | -0.010 | 0.030* | 0.003 | -0.062*** |
|  | (0.019) | (0.018) | (0.018) | (0.018) | (0.018) | (0.017) | (0.017) |
| **Marital Status** |  |  |  |  |  |  |  |
| Defactor (live together) | -0.052* | -0.039 | 0.002 | 0.023 | 0.013 | 0.053* | 0.021 |
|  | (0.031) | (0.031) | (0.030) | (0.030) | (0.030) | (0.028) | (0.029) |
| Defactor (live apart) | -0.080 | -0.087* | -0.048 | -0.076 | -0.053 | 0.000 | 0.012 |
|  | (0.049) | (0.048) | (0.048) | (0.047) | (0.046) | (0.043) | (0.045) |
| Married/Civil Union | -0.029 | -0.012 | -0.004 | -0.025 | 0.031 | 0.038 | -0.016 |
|  | (0.027) | (0.027) | (0.027) | (0.026) | (0.026) | (0.024) | (0.025) |
| Separated/Divorced | -0.029 | 0.046 | 0.060 | 0.020 | 0.090** | 0.093** | 0.066* |
|  | (0.043) | (0.042) | (0.042) | (0.042) | (0.041) | (0.038) | (0.040) |
| Widowed | 0.102 | -0.019 | 0.053 | -0.053 | -0.012 | 0.069 | 0.038 |
|  | (0.063) | (0.061) | (0.062) | (0.061) | (0.059) | (0.056) | (0.059) |
| Parent | 0.001 | 0.032 | -0.035* | -0.052*** | 0.018 | 0.032* | 0.049*** |
|  | (0.020) | (0.020) | (0.020) | (0.020) | (0.019) | (0.018) | (0.019) |
| **Highest education (base category – less than high school)** | | | | | | | |
| Completed high school | -0.065** | -0.110*** | -0.085*** | -0.114*** | -0.062** | -0.095*** | -0.083*** |
|  | (0.031) | (0.030) | (0.030) | (0.030) | (0.030) | (0.028) | (0.029) |
| TAFE or trade certificate | -0.071** | -0.093*** | -0.141*** | -0.125*** | -0.134*** | -0.112*** | -0.115*** |
|  | (0.029) | (0.028) | (0.028) | (0.028) | (0.027) | (0.025) | (0.027) |
| University | -0.154*** | -0.219*** | -0.220*** | -0.194*** | -0.205*** | -0.218*** | -0.134*** |
|  | (0.026) | (0.026) | (0.026) | (0.025) | (0.025) | (0.023) | (0.024) |
| **Employment (base - self employed)** | |  |  |  |  |  |  |
| Employed (wage/salary) | 0.049** | 0.027 | 0.053** | 0.038 | 0.082*** | 0.025 | -0.023 |
|  | (0.025) | (0.025) | (0.025) | (0.024) | (0.024) | (0.022) | (0.023) |
| Unemployed | 0.037 | 0.076 | 0.037 | 0.069 | 0.028 | -0.029 | -0.073 |
|  | (0.060) | (0.059) | (0.059) | (0.058) | (0.057) | (0.053) | (0.055) |
| Home duties | 0.127* | 0.005 | -0.081 | 0.013 | 0.009 | -0.100 | -0.076 |
|  | (0.072) | (0.071) | (0.071) | (0.070) | (0.069) | (0.064) | (0.067) |
| Student | 0.045 | -0.215*** | 0.016 | -0.037 | 0.025 | -0.087 | -0.095 |
|  | (0.067) | (0.063) | (0.066) | (0.065) | (0.064) | (0.059) | (0.062) |
| Retired | -0.018 | -0.021 | 0.033 | -0.014 | 0.032 | 0.003 | -0.017 |
|  | (0.034) | (0.034) | (0.034) | (0.033) | (0.033) | (0.031) | (0.032) |
| Unable to work | -0.008 | 0.173** | -0.026 | 0.099 | -0.006 | -0.088 | -0.138* |
|  | (0.087) | (0.086) | (0.085) | (0.084) | (0.083) | (0.077) | (0.081) |
| **Income (base - less than $10,000)** | |  |  |  |  |  |  |
| $10k- less than $20k | -0.039 | -0.124 | 0.069 | -0.070 | 0.126 | 0.135 | 0.126 |
|  | (0.102) | (0.100) | (0.100) | (0.099) | (0.097) | (0.090) | (0.094) |
| $20k - less than $30k | -0.024 | -0.213** | 0.024 | -0.145* | 0.004 | 0.042 | -0.051 |
|  | (0.091) | (0.089) | (0.089) | (0.088) | (0.087) | (0.080) | (0.084) |
| $30k - less than $40k | -0.113 | -0.218** | 0.024 | -0.055 | 0.015 | 0.061 | 0.048 |
|  | (0.090) | (0.088) | (0.088) | (0.087) | (0.086) | (0.080) | (0.083) |
| $40k - less than $50k | -0.041 | -0.165* | -0.020 | -0.068 | 0.018 | 0.083 | -0.001 |
|  | (0.088) | (0.086) | (0.086) | (0.085) | (0.084) | (0.078) | (0.081) |
| $50k - less than $60k | -0.037 | -0.024 | 0.109 | -0.044 | 0.060 | 0.115 | 0.117 |
|  | (0.086) | (0.084) | (0.084) | (0.083) | (0.082) | (0.076) | (0.080) |
| $60k - less than $80k | -0.092 | -0.173** | 0.010 | -0.088 | 0.090 | 0.086 | 0.079 |
|  | (0.083) | (0.081) | (0.081) | (0.080) | (0.079) | (0.073) | (0.076) |
| $80k - less than $100k | -0.048 | -0.183** | 0.018 | -0.055 | 0.070 | 0.074 | 0.080 |
|  | (0.083) | (0.081) | (0.081) | (0.080) | (0.079) | (0.073) | (0.076) |
| $100k - less than $125k | -0.078 | -0.151* | -0.004 | -0.032 | 0.029 | 0.096 | 0.054 |
|  | (0.083) | (0.081) | (0.081) | (0.080) | (0.079) | (0.073) | (0.077) |
| $1250k - less than $150k | -0.093 | -0.178** | -0.034 | -0.092 | 0.053 | 0.105 | 0.101 |
|  | (0.084) | (0.082) | (0.082) | (0.081) | (0.080) | (0.074) | (0.078) |
| $150k - less than $200k | -0.077 | -0.195** | 0.031 | -0.069 | 0.100 | 0.152** | 0.151** |
|  | (0.083) | (0.082) | (0.081) | (0.080) | (0.079) | (0.074) | (0.077) |
| $200k or more | -0.092 | -0.223*** | -0.022 | -0.093 | 0.086 | 0.140* | 0.171** |
|  | (0.083) | (0.082) | (0.082) | (0.081) | (0.079) | (0.074) | (0.077) |
| Prefer not to say | -0.087 | -0.159** | -0.014 | -0.111 | 0.002 | 0.058 | 0.032 |
|  | (0.080) | (0.079) | (0.079) | (0.078) | (0.077) | (0.071) | (0.074) |
| **Country of birth (base - Australia)** | |  |  |  |  |  |  |
| UK or New Zealand | -0.009 | -0.214*** | 0.003 | 0.013 | -0.036 | -0.047 | -0.126*** |
|  | (0.040) | (0.039) | (0.039) | (0.038) | (0.038) | (0.035) | (0.037) |
| Other | -0.248*** | -0.093 | -0.284*** | -0.016 | -0.363*** | -0.263*** | -0.203** |
|  | (0.094) | (0.093) | (0.092) | (0.091) | (0.090) | (0.084) | (0.087) |
| **Parent's Country of birth (base - both Australia)** | | |  |  |  |  |  |
| One in Australia | -0.006 | 0.017 | -0.016 | -0.040* | -0.001 | 0.010 | 0.038* |
|  | (0.024) | (0.023) | (0.023) | (0.023) | (0.023) | (0.021) | (0.022) |
| Both Overseas | 0.050** | 0.197*** | -0.038 | -0.102*** | 0.025 | -0.002 | 0.050** |
|  | (0.025) | (0.025) | (0.025) | (0.024) | (0.024) | (0.022) | (0.023) |
| ATSI | 0.203** | 0.132 | 0.097 | 0.106 | 0.142* | 0.133* | 0.104 |
|  | (0.089) | (0.088) | (0.087) | (0.086) | (0.085) | (0.079) | (0.082) |
| Disability or health issue | 0.045 | 0.081*** | -0.026 | 0.040 | 0.028 | 0.031 | 0.032 |
| (> 6 months) | (0.028) | (0.027) | (0.027) | (0.027) | (0.026) | (0.024) | (0.026) |
| Constant | -0.207 | 1.632*** | -0.913*** | 0.305 | -0.428* | 0.187 | 1.483*** |
|  | (0.289) | (0.124) | (0.283) | (0.279) | (0.236) | (0.256) | (0.267) |
| Observations | 14,813 | 14,813 | 14,813 | 14,813 | 14,813 | 14,813 | 14,813 |
| R-squared | 0.043 | 0.075 | 0.089 | 0.110 | 0.143 | 0.262 | 0.194 |
| LR test of age gender interaction (*p*-value) | 0.004 | 0.000 | 0.000 | 0.000 | 0.965 | 0.000 | 0.000 |
| Notes: Dependant variable modelled in each column is response to the respectively labelled statement below. Responses to each question are standardised to have a mean of zero and standard deviation of one. Age is included as a third order polynomial with average marginal effects presented. P-value of likelihood ratio (LR) test of interaction between age and gender presented in last row with interaction included if *p*-value < 0.05. Standard errors in parentheses. Level of statistical significance denoted by * (10%), ** (5%) and *** (1%).  (1): Most people in society think betting on sport is harmless.  (2): Most people in society bet on sport.  (3): Most people in my family think betting on sport is harmless.  (4): Most people in my family bet on sport.  (5): Most people in my friendship group think betting on sport is harmless.  (6): Most people in my friendship group bet on sport.  (7): Odds talk is common in discussions about sport with my friends and peers. | | | | | | | |

| Table 4: Full results of estimated models of perceptions of other’s attitudes and behaviours with respect to sports betting, focusing on number of non-sports and sports bets per year as explanatory variables. Table corresponds to Table 8 of paper. | | | | | | | |
| --- | --- | --- | --- | --- | --- | --- | --- |
| Variables | (1) | (2) | (3) | (4) | (5) | (6) | (7) |
| Number non-sport bets/year (100’s) | -0.008 | -0.005 | 0.111*** | 0.083*** | 0.106*** | 0.102*** | 0.088*** |
|  | (0.014) | (0.014) | (0.014) | (0.014) | (0.014) | (0.013) | (0.013) |
| Number sport bets/year (100’s) | 0.016 | 0.121*** | 0.216*** | 0.335*** | 0.203*** | 0.375*** | 0.300*** |
|  | (0.017) | (0.017) | (0.017) | (0.017) | (0.017) | (0.016) | (0.016) |
| Female | 0.120*** | 0.017 | 0.066*** | 0.141*** | -0.203*** | -0.423*** | -0.441*** |
|  | (0.019) | (0.018) | (0.019) | (0.018) | (0.018) | (0.017) | (0.017) |
| Age | -0.009*** | -0.013*** | -0.008*** | -0.007*** | -0.016*** | -0.020*** | -0.018*** |
|  | (0.001) | (0.001) | (0.001) | (0.001) | (0.001) | (0.001) | (0.001) |
| Regional | -0.015 | -0.018 | -0.000 | -0.016 | 0.025 | -0.002 | -0.064*** |
|  | (0.019) | (0.019) | (0.019) | (0.019) | (0.019) | (0.017) | (0.018) |
| **Marital Status** |  |  |  |  |  |  |  |
| Defacto (live together) | -0.038 | -0.032 | 0.014 | 0.048 | 0.033 | 0.066** | 0.039 |
|  | (0.032) | (0.031) | (0.032) | (0.031) | (0.031) | (0.029) | (0.030) |
| Defacto (live apart) | -0.060 | -0.078 | -0.023 | -0.041 | -0.016 | 0.008 | 0.038 |
|  | (0.050) | (0.049) | (0.050) | (0.049) | (0.048) | (0.045) | (0.047) |
| Married/Civil Union | -0.014 | -0.009 | 0.002 | -0.016 | 0.043 | 0.039 | -0.003 |
|  | (0.028) | (0.027) | (0.028) | (0.027) | (0.027) | (0.025) | (0.026) |
| Separated/Divorced | -0.019 | 0.046 | 0.087** | 0.040 | 0.123*** | 0.105*** | 0.080* |
|  | (0.044) | (0.043) | (0.044) | (0.043) | (0.043) | (0.040) | (0.041) |
| Widowed | 0.113* | -0.048 | 0.038 | -0.047 | 0.013 | 0.053 | 0.023 |
|  | (0.064) | (0.063) | (0.064) | (0.063) | (0.061) | (0.058) | (0.060) |
| Parent | 0.001 | 0.016 | -0.036* | -0.051** | 0.020 | 0.036* | 0.046** |
|  | (0.021) | (0.020) | (0.021) | (0.020) | (0.020) | (0.019) | (0.019) |
| **Highest education (base category – less than high school)** | | | | | | | |
| Completed high school | -0.054* | -0.099*** | -0.076** | -0.108*** | -0.057* | -0.090*** | -0.079*** |
|  | (0.032) | (0.031) | (0.032) | (0.031) | (0.031) | (0.029) | (0.030) |
| TAFE or trade certificate | -0.062** | -0.094*** | -0.136*** | -0.114*** | -0.132*** | -0.100*** | -0.103*** |
|  | (0.029) | (0.029) | (0.029) | (0.029) | (0.028) | (0.027) | (0.027) |
| University | -0.138*** | -0.215*** | -0.243*** | -0.205*** | -0.228*** | -0.237*** | -0.141*** |
|  | (0.027) | (0.026) | (0.027) | (0.026) | (0.026) | (0.024) | (0.025) |
| **Employment (base - self employed)** | |  |  |  |  |  |  |
| Employed (wage/salary) | 0.065** | 0.030 | 0.056** | 0.036 | 0.088*** | 0.025 | -0.024 |
|  | (0.026) | (0.025) | (0.026) | (0.025) | (0.025) | (0.023) | (0.024) |
| Unemployed | 0.053 | 0.060 | 0.038 | 0.076 | 0.011 | -0.049 | -0.102* |
|  | (0.062) | (0.061) | (0.062) | (0.061) | (0.060) | (0.056) | (0.058) |
| Home duties | 0.102 | 0.010 | -0.124* | -0.022 | -0.010 | -0.127* | -0.107 |
|  | (0.073) | (0.072) | (0.073) | (0.072) | (0.071) | (0.067) | (0.068) |
| Student | 0.094 | -0.146** | -0.008 | -0.059 | 0.010 | -0.139** | -0.115* |
|  | (0.069) | (0.067) | (0.069) | (0.067) | (0.066) | (0.062) | (0.064) |
| Retired | -0.014 | -0.024 | 0.017 | -0.023 | 0.022 | -0.008 | -0.022 |
|  | (0.035) | (0.034) | (0.035) | (0.034) | (0.034) | (0.032) | (0.033) |
| Unable to work | -0.003 | 0.183** | -0.049 | 0.081 | -0.015 | -0.086 | -0.150* |
|  | (0.088) | (0.087) | (0.088) | (0.087) | (0.085) | (0.080) | (0.082) |
| **Income (base - less than $10,000)** | |  |  |  |  |  |  |
| $10k- less than $20k | -0.003 | -0.153 | 0.139 | -0.033 | 0.131 | 0.159* | 0.171* |
|  | (0.105) | (0.103) | (0.105) | (0.103) | (0.101) | (0.095) | (0.098) |
| $20k - less than $30k | 0.008 | -0.260*** | 0.096 | -0.108 | 0.024 | 0.045 | -0.041 |
|  | (0.093) | (0.092) | (0.093) | (0.092) | (0.090) | (0.085) | (0.087) |
| $30k - less than $40k | -0.099 | -0.244*** | 0.100 | -0.010 | 0.053 | 0.096 | 0.081 |
|  | (0.092) | (0.091) | (0.092) | (0.091) | (0.089) | (0.084) | (0.086) |
| $40k - less than $50k | -0.038 | -0.192** | 0.039 | -0.039 | 0.033 | 0.096 | -0.003 |
|  | (0.090) | (0.088) | (0.090) | (0.088) | (0.087) | (0.082) | (0.084) |
| $50k - less than $60k | -0.018 | -0.044 | 0.176** | -0.017 | 0.076 | 0.126 | 0.131 |
|  | (0.088) | (0.086) | (0.088) | (0.086) | (0.085) | (0.080) | (0.082) |
| $60k - less than $80k | -0.093 | -0.197** | 0.092 | -0.037 | 0.117 | 0.110 | 0.093 |
|  | (0.085) | (0.083) | (0.084) | (0.083) | (0.082) | (0.077) | (0.079) |
| $80k - less than $100k | -0.040 | -0.209** | 0.108 | -0.009 | 0.103 | 0.107 | 0.107 |
|  | (0.085) | (0.083) | (0.084) | (0.083) | (0.082) | (0.077) | (0.079) |
| $100k - less than $125k | -0.073 | -0.177** | 0.083 | 0.016 | 0.059 | 0.131* | 0.088 |
|  | (0.085) | (0.083) | (0.085) | (0.083) | (0.082) | (0.077) | (0.079) |
| $1250k - less than $150k | -0.088 | -0.211** | 0.056 | -0.043 | 0.089 | 0.136* | 0.126 |
|  | (0.086) | (0.084) | (0.086) | (0.084) | (0.083) | (0.078) | (0.080) |
| $150k - less than $200k | -0.080 | -0.223*** | 0.119 | -0.015 | 0.133 | 0.195** | 0.181** |
|  | (0.085) | (0.083) | (0.085) | (0.084) | (0.082) | (0.077) | (0.079) |
| $200k or more | -0.098 | -0.256*** | 0.079 | -0.021 | 0.128 | 0.199** | 0.210*** |
|  | (0.085) | (0.084) | (0.085) | (0.084) | (0.083) | (0.077) | (0.080) |
| Prefer not to say | -0.087 | -0.176** | 0.055 | -0.073 | 0.023 | 0.081 | 0.053 |
|  | (0.082) | (0.081) | (0.082) | (0.081) | (0.080) | (0.075) | (0.077) |
| **Country of birth (base - Australia)** | |  |  |  |  |  |  |
| UK or New Zealand | -0.013 | -0.200*** | 0.010 | 0.015 | -0.021 | -0.033 | -0.123*** |
|  | (0.040) | (0.039) | (0.040) | (0.039) | (0.039) | (0.036) | (0.037) |
| Other | -0.236** | -0.094 | -0.323*** | -0.029 | -0.380*** | -0.285*** | -0.225** |
|  | (0.095) | (0.093) | (0.095) | (0.093) | (0.092) | (0.086) | (0.089) |
| **Parent's Country of birth (base - both Australia)** | | |  |  |  |  |  |
| One in Australia | -0.010 | -0.003 | -0.021 | -0.055** | -0.009 | 0.002 | 0.026 |
|  | (0.024) | (0.024) | (0.024) | (0.024) | (0.023) | (0.022) | (0.022) |
| Both Overseas | 0.060** | 0.186*** | -0.046* | -0.108*** | 0.012 | -0.012 | 0.052** |
|  | (0.026) | (0.025) | (0.026) | (0.025) | (0.025) | (0.023) | (0.024) |
| ATSI | 0.212** | 0.185** | 0.090 | 0.116 | 0.160* | 0.143* | 0.139 |
|  | (0.093) | (0.091) | (0.093) | (0.091) | (0.090) | (0.084) | (0.087) |
| Diability or health issue | 0.049* | 0.080*** | -0.025 | 0.034 | 0.019 | 0.024 | 0.029 |
| (> 6 months) | (0.028) | (0.028) | (0.028) | (0.028) | (0.027) | (0.026) | (0.026) |
| Constant | -0.257 | 0.525* | -0.787*** | 0.503* | -0.322 | 0.504* | 1.575*** |
|  | (0.300) | (0.294) | (0.299) | (0.294) | (0.246) | (0.272) | (0.280) |
| Observations | 14,293 | 14,293 | 14,293 | 14,294 | 14,293 | 14,293 | 14,293 |
| R-squared | 0.043 | 0.075 | 0.053 | 0.067 | 0.118 | 0.215 | 0.176 |
| LR test of age gender interaction (*p*-value) | 0.006 | 0.001 | 0.004 | 0.006 | 0.285 | 0.000 | 0.000 |
| Notes: Dependant variable modelled in each column is response to the respectively labelled statement below. Responses to each question are standardised to have a mean of zero and standard deviation of one. Age is included as a third order polynomial with average marginal effects presented. P-value of likelihood ratio (LR) test of interaction between age and gender presented in last row with interaction included if *p*-value < 0.05. Standard errors in parentheses. Level of statistical significance denoted by * (10%), ** (5%) and *** (1%).  (1): Most people in society think betting on sport is harmless.  (2): Most people in society bet on sport.  (3): Most people in my family think betting on sport is harmless.  (4): Most people in my family bet on sport.  (5): Most people in my friendship group think betting on sport is harmless.  (6): Most people in my friendship group bet on sport.  (7): Odds talk is common in discussions about sport with my friends and peers. | | | | | | | |
